# A Comparative Analysis of Drug-Induced Hepatotoxicity in Clinically Relevant Situations

**DOI:** 10.1371/journal.pcbi.1005280

**Published:** 2017-02-02

**Authors:** Christoph Thiel, Henrik Cordes, Lorenzo Fabbri, Hélène Eloise Aschmann, Vanessa Baier, Ines Smit, Francis Atkinson, Lars Mathias Blank, Lars Kuepfer

**Affiliations:** 1 Institute of Applied Microbiology (iAMB), Aachen Biology and Biotechnology (ABBt), RWTH Aachen University, Worringerweg 1, Aachen, Germany; 2 European Molecular Biology Laboratory, European Bioinformatics Institute (EMBL-EBI), Wellcome Genome Campus, Hinxton, Cambridge, United Kingdom; Tufts University, UNITED STATES

## Abstract

Drug-induced toxicity is a significant problem in clinical care. A key problem here is a general understanding of the molecular mechanisms accompanying the transition from desired drug effects to adverse events following administration of either therapeutic or toxic doses, in particular within a patient context. Here, a comparative toxicity analysis was performed for fifteen hepatotoxic drugs by evaluating toxic changes reflecting the transition from therapeutic drug responses to toxic reactions at the cellular level. By use of physiologically-based pharmacokinetic modeling, in vitro toxicity data were first contextualized to quantitatively describe time-resolved drug responses within a patient context. Comparatively studying toxic changes across the considered hepatotoxicants allowed the identification of subsets of drugs sharing similar perturbations on key cellular processes, functional classes of genes, and individual genes. The identified subsets of drugs were next analyzed with regard to drug-related characteristics and their physicochemical properties. Toxic changes were finally evaluated to predict both molecular biomarkers and potential drug-drug interactions. The results may facilitate the early diagnosis of adverse drug events in clinical application.

## Author Summary

Liver toxicity may occur at drug levels above the therapeutic range and is thus a crucial problem in clinical care. However, the cellular changes induced by drug administration of therapeutic and toxic doses in humans are still not well understood. We here coupled patient-specific drug concentration-time profiles following oral administration of therapeutic and toxic doses with in vitro drug response data to predict toxic changes that quantitatively reflect the transition from desired drug effects to undesired toxic reactions. These toxic changes were comparatively evaluated for fifteen hepatotoxic drugs to identify subsets of drugs, which show similar drug effects on key cellular processes, functional classes of genes, and individual genes, respectively. In addition, analyzing toxic changes for individual genes allowed the prediction of molecular biomarkers and potential drug-drug interactions. Our results may hence support the early diagnosis of liver toxicity in clinical care in the future and may, moreover, help to assess potential risks of drug combination therapies.

## Introduction

Drug-induced hepatotoxicity poses a significant problem in drug development and public health [1,2]. Extensive drug exposure due to overdosing or patient idiosyncrasy may lead to hepatotoxic effects such as drug-induced steatosis or cholestasis [3,4,5]. Such adverse events may even be aggravated through drug-drug interactions (DDIs) during patient co-medication leading to additive, synergistic, or antagonist drug effects [6,7,8,9].

Understanding the molecular mechanisms underlying the transition from desired drug effects to adverse events induced by therapeutic and toxic doses, respectively, is of general importance for both clinical diagnostics and curative intervention strategies [10]. In this regard, robust clinical biomarkers may significantly improve patient safety and health [11,12,13,14,15] by the initial identification of cellular mechanisms indicating drug toxicity in order to implement appropriate interventions at an early stage [16,17,18,19]. Comparatively analyzing cellular responses following the transition from therapeutic to toxic doses supports the identification of molecular biomarkers and would clearly help to investigate to what extent specific drugs similarly contribute to characteristic toxicological processes and, furthermore, to find out potential interactions between those drugs, which might act on a mutual target gene.

**Fig 1:**
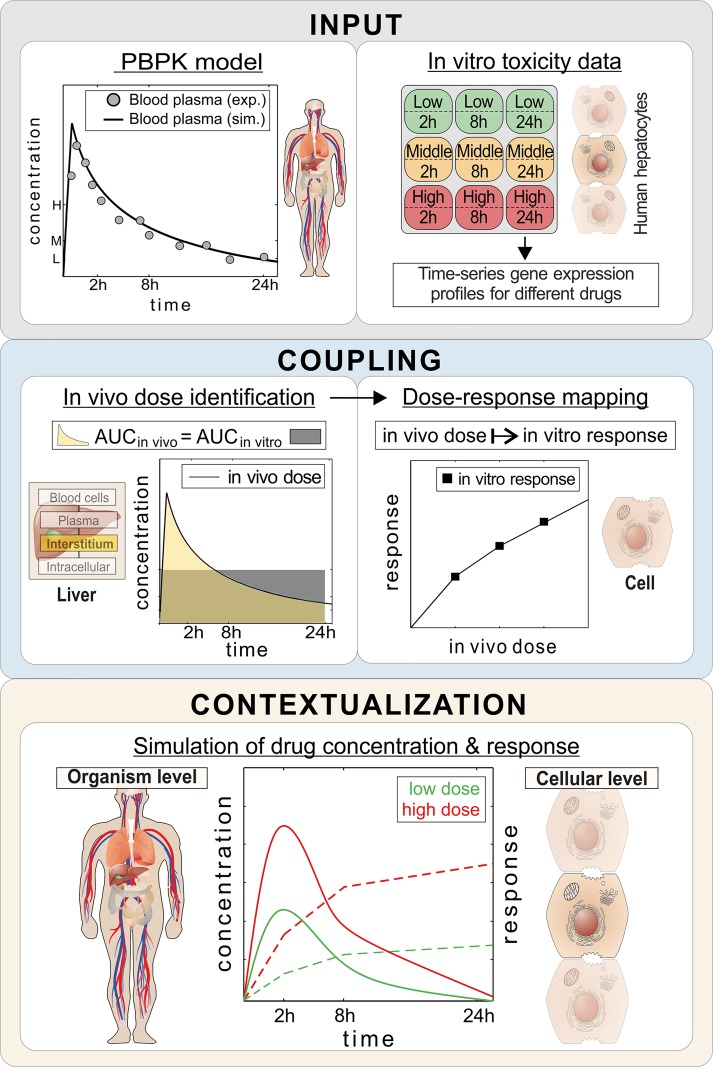
PBPK-based in vivo contextualization of in vitro toxicity data (PICD). INPUT: At the organism level, PBPK models are developed for specific drugs. At the cellular level, in vitro response data of compound-treated primary hepatocytes are analyzed (28). COUPLING: In vivo doses are identified, which are directly related to in vitro drug exposure (AUC_in vivo_ = AUC_in vitro_). Time-dependent dose-response curves are built by mapping in vivo doses to in vitro responses. CONTEXTUALIZATION: By use of the time-dependent dose-response curves drug responses over time are predicted for PK profiles simulated for different doses. (Illustration of cells and parts of human body adapted with permission [75], https://creativecommons.org/licenses/by/4.0/)

A comparative study of molecular responses in human cell lines in the face of therapeutic and toxic doses for a set of known hepatotoxic drugs could be used to better characterize drug-induced toxicity. A severe drawback of such in vitro analyses, however, is often the limited translatability to the in vivo situation in patients in actual clinical practice. Recently, we have developed an integrative multiscale approach called PICD for the in vivo contextualization of in vitro toxicity data based on physiologically-based pharmacokinetic (PBPK) modeling, which significantly supports translations to an in vivo situation in patients (Fig 1) [20]. Importantly, PBPK modeling aims for a mechanistic representation of absorption, distribution, metabolism, and elimination (ADME) processes governing drug pharmacokinetics (PK) within the human body. Since PBPK models include a large amount of mechanistic information, these models are well-suited for extrapolations to different treatment scenarios.

**Fig 2:**
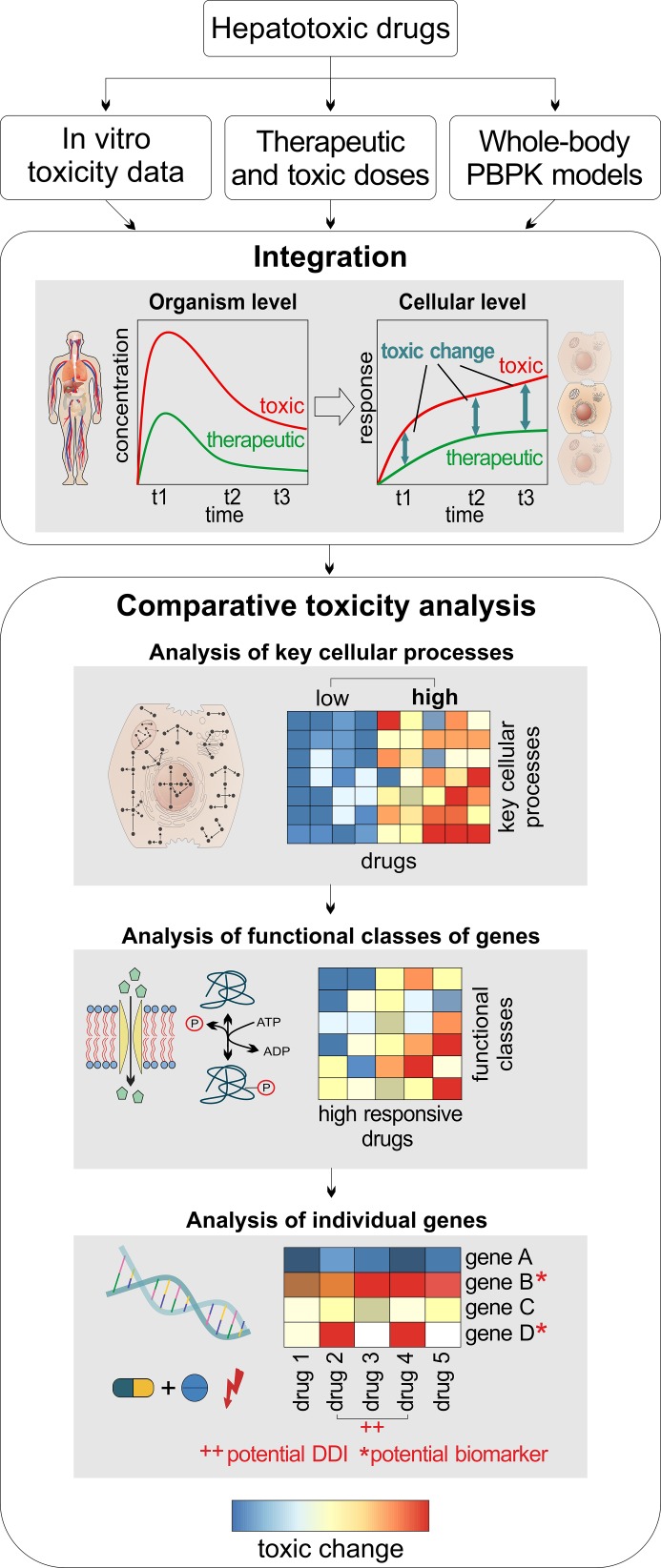
General workflow. For a set of hepatotoxic drugs, in vitro toxicity data from Open TG-GATEs [21] were analyzed, therapeutic and toxic doses were identified in the literature, and whole-body PBPK models were developed and validated. Toxic changes were then predicted at different timepoints (2 h, 8 h, 24 h) by comparing cellular response following drug administration of therapeutic and toxic doses and were subsequently evaluated with regard to key cellular processes, functional classes of genes, and individual genes, respectively. (Illustration of cells and parts of human body adapted with permission [75], https://creativecommons.org/licenses/by/4.0/)

The main goal of this study was the analysis of drug-induced toxicity following administration of therapeutic and toxic doses of different hepatotoxicants in humans. Thus, toxic changes reflecting drug-induced toxicity during the transition from therapeutic to toxic doses were comparatively evaluated for fifteen hepatotoxicants to quantitatively identify subsets of drugs, which share similar perturbations on (i) key cellular processes, (ii) functional classes of genes, and (iii) individual genes (Fig 2). To predict drug responses in clinically relevant situations following administration of therapeutic and toxic doses, PBPK-based in vivo contextualization of in vitro toxicity data (PICD) (Fig 1) was applied on a set of fifteen known hepatotoxic drugs: acetaminophen (APAP), amiodarone (AD), azathioprine (AZA), cyclophosphamide (CPA), cyclosporine A (CSA), diclofenac (DFN), erythromycin (ERY), flutamide (FT), haloperidol (HPL), isoniazid (INH), phenobarbital (PB), phenytoin (PHE), rifampicin (RIF), simvastatin (SST), valproic acid (VPA). The drugs were selected based on pharmaceutical and chemical diversity, physicochemical properties, availability of in vitro toxicity data and experimental drug concentration-time profiles as well as concern for drug-induced liver injury (DILI) (S1 Table). Transcriptome data obtained in primary human hepatocytes from Open TG-GATEs [21] was used as in vitro toxicity data at the cellular level, while human PBPK models were developed at the organism level. In the comparative toxicity analysis, toxic changes were evaluated in three different analyses (Fig 2). In the first analysis, toxic changes between the fifteen hepatotoxic drugs were investigated for a large number of key cellular processes (S2 Table). In the second analysis, toxic changes calculated for different functional classes of genes were evaluated for a subset of key cellular processes strongly perturbed by an identified set of high-responsive drugs. In the third analysis, toxic changes were evaluated for a set of individual genes thereby quantitatively discovering molecular biomarkers and potential DDIs for the high-responsive drugs.

## Results

### Human whole-body PBPK models

**Fig 3:**
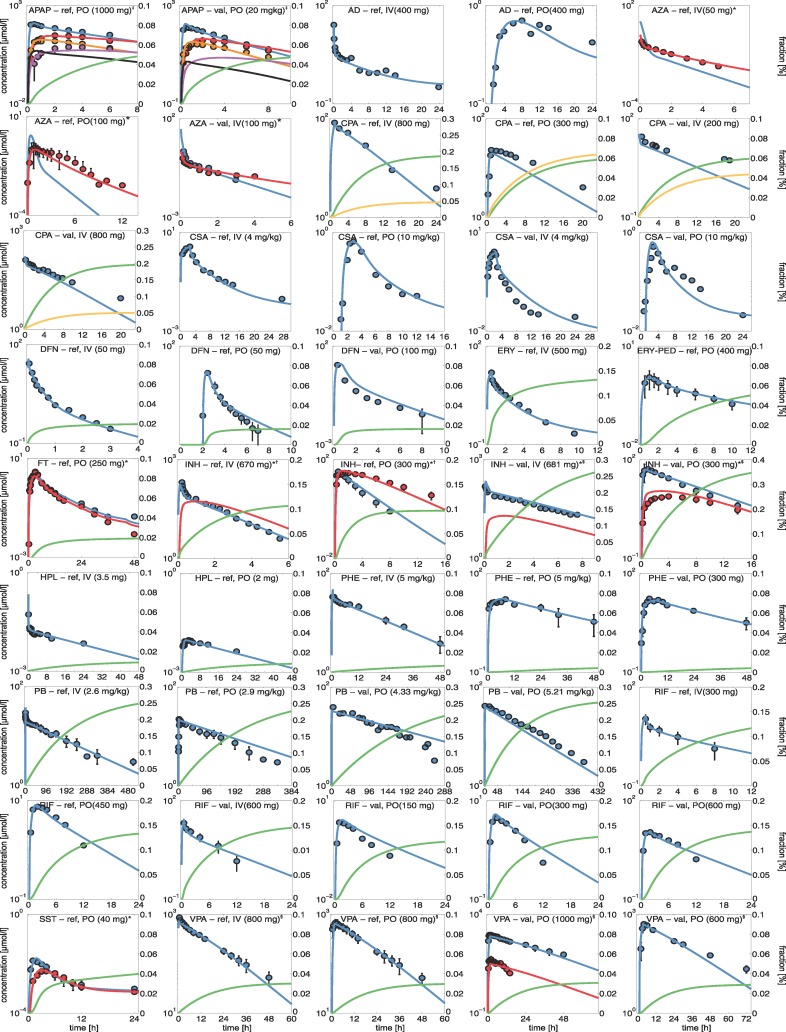
Human PBPK models. Simulated concentration-time curves (lines) for all parent drugs (blue) were assessed with experimental PK profiles (circles) used for developing reference (ref.) or validated (val.) human PBPK models. Drugs were either administered orally (PO) or intravenously (IV). Additionally, renal (green) and biliary (dark yellow) excretion rates were simulated. * Primary metabolites (red) 6-MP, 2-hydroxy-FT, acetyl-INH, and SST-acid; ‡ APAP-glucuronide (red), APAP-sulfate (orange), APAP-cysteine (purple), and NAPQI (black); † Rapid metabolizer; $ Slow metabolizer; § Unbound plasma concentrations (red).

Whole-body PBPK models were initially established for a set of fifteen hepatotoxic drugs and were carefully validated with human experimental data from literature (S3 Table). The validated PBPK models served as input for PICD (PBPK-based in vivo contextualization of in vitro toxicity data) [20] to quantify in vivo drug responses induced by therapeutic and toxic doses administered in humans. Physicochemical properties, plasma protein binding, and lipophilicity of the different drugs and their metabolites were obtained from literature and were used to develop the reference PBPK model for intravenous administration in humans (Table 1). Key metabolic reactions and active drug transport were integrated into the human PBPK models to represent the main ADME processes (S4 Table). Relative tissue-specific abundances of relevant enzymes and transporters were estimated using tissue-specific gene expression data [22]. To describe the elimination of the drugs and their metabolites, renal and biliary clearance processes were incorporated into the human PBPK models (S5 Table) and parametrized such that simulations are in agreement with experimental observations (S5 Table). After model establishment, the simulated drug concentrations in plasma showed an excellent agreement with in vivo PK data measured in humans (Fig 3, S1 Fig).

**Table 1:** Physicochemical drug properties used in the developed PBPK models. Molecular weight (MW), octanol/water partition coefficient (logP), fraction unbound (Fu), acid dissociation constant (pKa), and water solubility used in the developed PBPK models. Molecular weights are taken from DrugBank [23], references for other properties were explicitly presented. In some cases, logP and Fu values were slightly adjusted to best describe the experimental data. NAPQI, N-acetyl-p-benzoquinone imine; 6-MP, 6-mercaptopurine; ERY-PED, erythromycin ethylsuccinate.

ID	Drug/Metabolite	MW [g/mol]	logP	Reference	Fu	Reference	Compound type	pkA	Reference	Water Solubility [mg/l]	Reference
1	APAP	151.16	0.33	[23]	0.81	[23]	Acid	9.38	[23]	14000.00	[23]
1	APAP-cysteine	254.31	0.40	[23]	0.6	*	[Acid, base]	[1.93, 9.09]	[23]	337.00	[23]
1	APAP-glucuronide	327.29	-0.98	[23]	0.98	*	Acid	3.17	[23]	27700.00	[23]
1	APAP-sulfate	231.23	-0.52	[23]	0.80	*	[Acid, base]	[-2.16,14.65]	[24]	1540.00	[23]
1	NAPQI	149.15	0.1	[23]	0.02	[25]	Neutral	-	[24]	987.00	[23]
2	AD	645.31	4.67	[26]	0.0032	[27]	Base	6.56	[28]	4.76	[23]
3	6-MP	152.18	1.85	[29]	0.81	[23]	[Acid, base]	[9.50, 2.99]	[23]	68500.00	[23]
3	AZA	277.26	0.10	[23]	0.70	[23]	Base	7.87	[23]	1007.00	[23]
4	CPA	261.09	0.80	[23]	0.80	[23]	Acid	6.00	[30]	30000.00	[23]
5	CSA	1202.61	3.88	[31]	0.09	[32]	Base	11.83	[23]	5.81	[23]
6	DFN	296.15	4.10	[23]	0.0035	[33]	Acid	4.15	[23]	2.37	[23]
7	ERY	733.93	3.06	[23]	0.18	[23]	Base	8.88	[23]	2000.00	[23]
7	ERY-PED	862.06	3.84	*	0.18	[23]	Acid	7.10	[34]	2000.00	*
8	2-hydroxy FT	292.21	2.08	[23]	0.028	[35]	Acid	3.80	[23]	5.56	[23]
8	FT	276.21	3.05	[23]	0.052	[35]	Base	13.17	[23]	9.45	[23]
9	HPL	375.86	3.60	[23]	0.06	[23]	Base	8.66	[23]	14.00	[23]
10	Acetyl-INH	179.18	-0.90	[36]	0.90	*	[Acid, base]	[6.77, 3.02]	[36]	1770.00	[36]
10	INH	137.14	-0.67	[23]	0.90	[23]	[Acid, base]	[13.61, 3.35]	[23]	140000.00	[23]
11	PB	232.24	0.13	[23]	0.57	[23]	Acid	7.30	[23]	1110.00	[23]
12	PHE	252.27	2.26	[23]	0.098	[37]	Acid	8.33	[23]	32.00	[23]
13	RIF	822.94	2.93	[23]	0.195	[38]	Acid	1.70	[23]	1400.00	[23]
14	SST	418.57	4.68	[23]	0.03	[39]	Neutral	-	[40]	0.76	[40]
14	SST-acid	436.58	4.3	[36]	0.056	[23]	Acid	4.31	[40]	11.00	[40]
15	Hydroxyl-VPA	160.21	1.42	[36]	0.04	*	Acid	4.81	[36]	45100.00	[36]
15	VPA	114.21	1.85	[23]	0.04	[41]	Acid	5.14	[23]	1300.00	[23]
15	VPA-β-glucuronide	320.33	0.85	[36]	0.04	*	Acid	3.41	[36]	22200.00	[23]

* adjusted/adopted from parent drug

To validate the established reference PBPK models, experimental PK data from different studies, which had not been used during initial model establishment, were next used to simulate concentration-time profiles for additional dosage regimens and patient subgroups (Fig 3, S1 Fig). Notably, model parameters were left unchanged for model validation except the intestinal permeability where the initial reference value was slightly adjusted in some cases, when the drug was given orally (S6 Table).

The PBPK model parameters (Table 1, S4 Table, S5 Table, S6 Table, S7 Table) together with the specific information about the clinical studies (S3 Table) are sufficient to fully reproduce all developed human PBPK models due to the large degree of prior information, which is already included in PBPK models. Importantly, the validated PBPK models allow accurate simulations for different dose levels, including therapeutic or toxic doses, since potential non-linearity’s in ADME processes are implicitly represented through the underlying model structure.

### Prediction of in vivo drug responses for humans by integrating in vitro toxicity data into whole-body PBPK models

**Fig 4:**
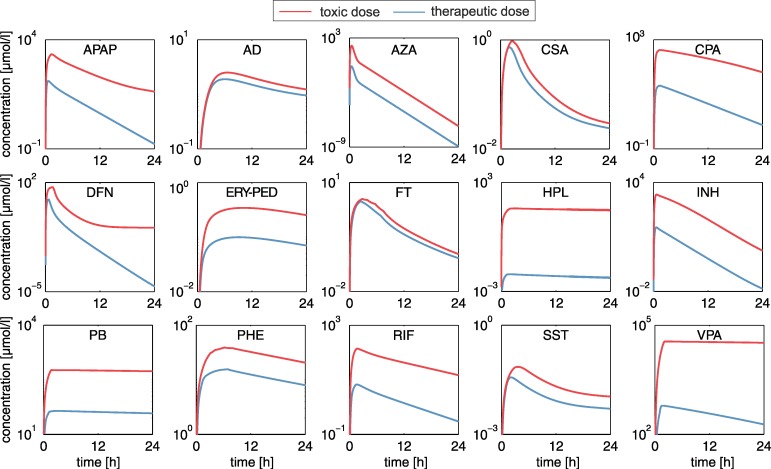
Therapeutic and toxic PK profiles. Plasma concentration-time profiles simulated for drug administration of the therapeutic (blue) and the toxic (red) doses in humans (S3 Table, S8 Table).

To analyze and compare drug-induced hepatotoxicity of the fifteen drugs within a patient context, toxic changes reflecting the transition from desired drug effects to adverse events were considered by predicting time-dependent in vivo responses for humans following drug administration of therapeutic and toxic doses. In vitro toxicity data from Open TG-GATEs [21] measured in primary human hepatocytes for the fifteen hepatotoxic drugs were therefore analyzed. Toxicity lists from QIAGENs Ingenuity Pathway Analysis (IPA, QIAGEN Redwood City, www.qiagen.com/ingenuity) were used to represent biological processes associated to critical toxicological responses and are further referred to as ‘key cellular processes’ (S2 Table). Drug concentration-time profiles were simulated for therapeutic and toxic doses identified in literature (Fig 4, S8 Table) by using the developed human PBPK models (Fig 3). PICD was next applied to translate in vitro findings to an in vivo situation within patients.

In brief, the basic concept of PICD is the identification of in vivo doses such that the simulated drug exposure in the interstitial space of the liver is equal to the in vitro drug exposure of the assay. The identified in vivo doses were mapped to the in vitro toxicity data in order to describe time-dependent in vivo drug responses at different dose levels (Fig 1) [20]. After applying PICD, in vivo drug responses for humans induced by therapeutic and toxic doses could be predicted for the considered key cellular processes.

### Validation of predicted in vivo drug responses in rats

To validate the predictive accuracy of the PICD-based in vitro-in vivo translation, PICD was next applied for rats, because in vivo data were only available for rats but not for humans [21]. Since PICD requires PBPK models as input at the organism level, rat PBPK models were developed by applying cross-species extrapolation thereby taking into account species-specific differences to extrapolate PK profiles between humans and rats [42]. In vitro toxicity data measured in rat hepatocytes [21] were then translated to an in vivo situation by applying PICD on rat PBPK models. For each drug, significantly perturbed key cellular processes for rats were identified (S1 Dataset) and correspondent in vivo drug responses were subsequently predicted for the relevant doses that have been administered in the in vivo rat study [21]. Finally, predicted drug responses were correlated with in vivo observations.

**Fig 5:**
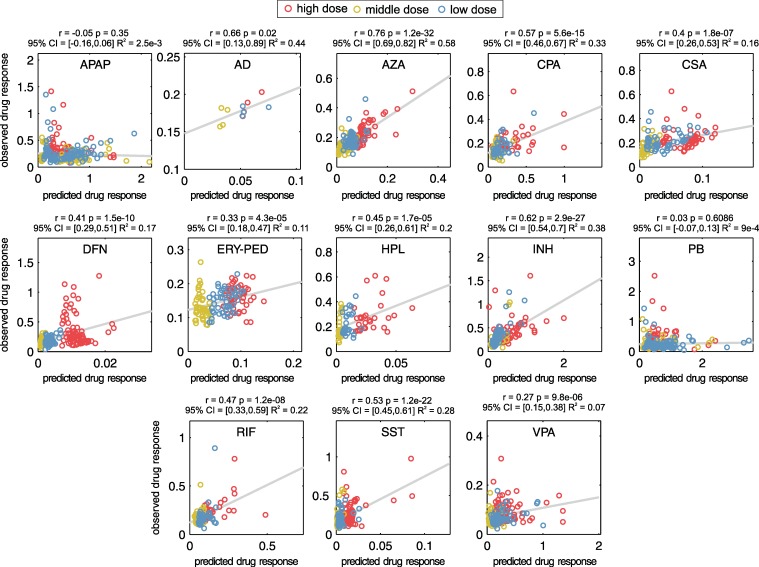
Validation of predicted drug response with in vivo measurements in rats. In vivo drug responses of significantly perturbed key cellular processes (S1 Dataset) predicted for the three doses used in the in vivo rat study were compared to observations measured in vivo [21].

Correlation analyses between predicted and observed in vivo rat data revealed moderate correlations (r = 0.27–0.76, p < 0.05, R^2^ = 0.07–0.58) (Fig 5) for all drugs apart from PB (r = 0.03, p = 0.6, 95% confidence interval (95% CI) = [-0.07, 0.13], R^2^ = 9.4E-4) and APAP (r = -0.05, p = 0.35, 95% CI = [-0.16, 0.06], R^2^ = 0.0025) (Fig 5). These correlations obtained in a preparatory proof-of-concept analysis in rats are mostly statistically significant albeit not that strong in some cases.

### Comparative toxicity analysis

In the comparative toxicity analysis, drug-induced hepatotoxicity was investigated within a patient context to identify subsets of drugs, which share similar perturbation on key cellular processes, functional classes of genes, as well as individual genes. Toxic changes reflecting the transition from desired drug effects to adverse events were therefore calculated for humans and were compared among the set of fifteen hepatotoxic drugs (S1 Table). The application of PICD allowed predicting time-dependent drug responses of therapeutic and toxic doses in an in vivo context [20]. Note that all in vivo drug response values predicted for the toxic dose were higher than the respective values predicted for the therapeutic dose, such that all toxic changes are positive.

### Analysis of key cellular processes

**Fig 6:**
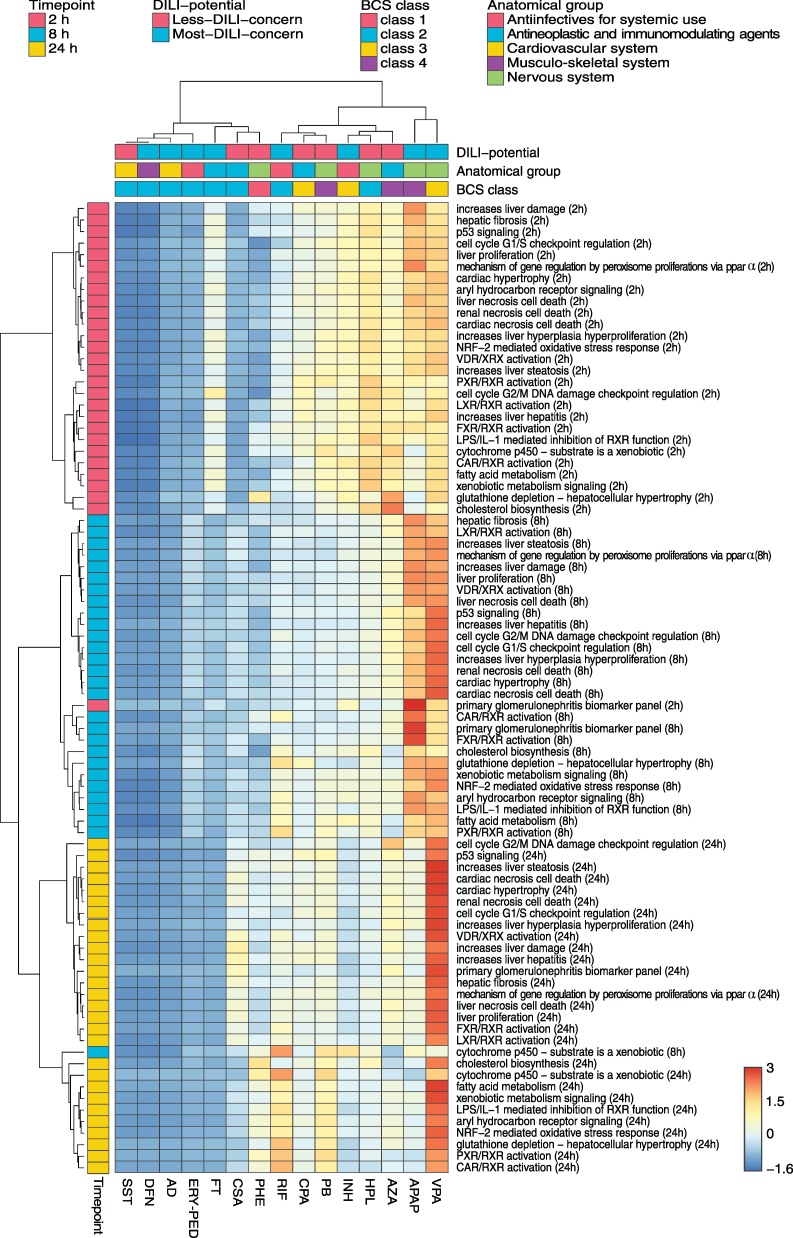
Comparative toxicity analysis of key cellular processes in humans. Toxic changes in perturbed key cellular processes (S2 Table) were calculated for fifteen hepatotoxic drugs at 2 h, 8 h and 24 h. The drugs were annotated with their respective DILI-potential, the BCS class and the target organ or system (S1 Table). The dendrograms were constructed using the Ward's minimum variance algorithm. The color scale depicts normalized toxic changes. The heatmap was visualized by use of the web tool ClustVis [76]. Row-normalization is performed by subtracting the mean and by dividing the respective standard deviation.

In the first analysis, toxic changes calculated for humans were evaluated at three different time points (2 h, 8 h, and 24 h) for key cellular processes that were significantly overrepresented in at least one third of the drugs (Fig 6, S2 Dataset).

Hierarchical clustering identified three major groups, which showed a clear separation between the considered timepoints (Fig 6). This observation was also confirmed by applying a principal component analysis (S2 Fig). Interestingly, low toxic changes were observed for SST, DFN, and AD at all timepoints. In contrast, high toxic changes (e.g., for genes involved in liver damage, liver hepatitis, liver steatosis, and liver proliferation) were found already at 2 h for HPL, APAP, VPA, AZA, and INH. AZA and VPA further depicted a high impact on genes involved in hepatocellular hypertrophy resulting in glutathione depletion (Fig 6). At 8 h, VPA and APAP revealed substantially high activity on several key cellular processes in particular on liver proliferation, liver damage, and liver hyperplasia (Fig 6). Furthermore, the regulation of the cell cycle G2/M DNA damage checkpoint, on the one hand, as well as the activation of the FXR/RXR and CAR/RXR heterodimers, on the other hand, were clearly perturbed after 8 h by APAP and AZA, respectively (Fig 6). At 24 h, VPA primarily affected all considered key cellular processes (Fig 6).

Hierarchical clustering was next performed to classify the fifteen hepatotoxic drugs according to similar hepatotoxic potential. Two main clusters could be identified where the first cluster (SST, DFN, AD, ERY, FT, CSA, and PHE) basically showed a lower response on key cellular processes than the second one (RIF, CPA, PB, INH, HPL, AZA, APAP, and VPA) (p = 9E-66, 95% confidence interval for the difference between the two groups (95% CI) = [0.079, 0.098], two-sample t-test). The low-responsive group was further subclustered into SST, DFN, AD and ERY, on the one hand, and into FT, CSA and PHE, on the other hand. The high-responsive group could be further subdivided into three smaller sub clusters: the first consists of RIF, CPA, and PB; the second of AZA, HPL, and INH; the third only of APAP and VPA.

The hierarchical clustering results were further analyzed to test whether the low- and high-responsive drugs could be attributed to (i) pharmacokinetic parameters, (ii) drug permeability and solubility properties (BCS class) [43], (iii) their target organ or system (anatomical main group), or (iv) their concern for causing DILI (DILI-potential) (S1 Table). Results from this analysis show that the low-responsive drugs were significantly higher bound to plasma proteins (p = 0.0098, 95% CI = [0.15, 0.74], two-sample t-test), and were more lipophilic (p = 0.0013, 95% CI = [1.24, 4.05], two-sample t-test) (Table 1). Investigating the solubility properties between both groups revealed no significant difference (p = 0.21, 95% CI = [-16964.77, 63585.33], two-sample t-test) (Table 1). Interestingly, toxic changes calculated for both groups were independent from both the ratio of toxic and therapeutic doses (p = 0.33, 95% CI = [-1509.31, 3929.78], two-sample t-test) (S3 Table, S8 Table) and from the ratio of correspondent area under the curve values (AUC_0-24h_: p = 0.35, 95% CI = [-2341.65, 5798.28], two-sample t-test) (Fig 4).

Comparison of both main clusters also showed no clear distinction of annotated DILI-potentials (Fig 6) with regard to drug-specific characteristics, which was also observed for the assigned severity scores (p = 0.7, 95% CI = [-2.12, 3.09], two-sample t-test) (Fig 6, S1 Table). Contrarily, the drugs classified as BCS class 3 (low permeability, high solubility) and 4 (low permeability, low solubility) tended to belong to the high-responsive drugs while the low-responsive group was enriched with drugs annotated with BCS class 2 (low solubility, high permeability). Furthermore, drugs were not clearly separable based on their target organ or system (Fig 6). Nevertheless, drugs acting on the cardiovascular system (SST and AD) or on the musculo-skeletal system (DFN) were clustered together, while anti-infective and drugs acting on the nervous system were rather assigned to the high-responsive group (Fig 6, S1 Table).

### Analysis of functional classes of genes

Next, toxic changes were analyzed at the functional level to quantitatively describe to what extent single drugs or subset of drugs perturbed different functional classes of genes, such as kinases or metabolic enzymes, associated to key cellular processes. Note that only the previously identified set of the high-responsive drugs and a subset of key cellular processes, which were strongly induced by these drugs, were here considered in the following.

**Fig 7:**
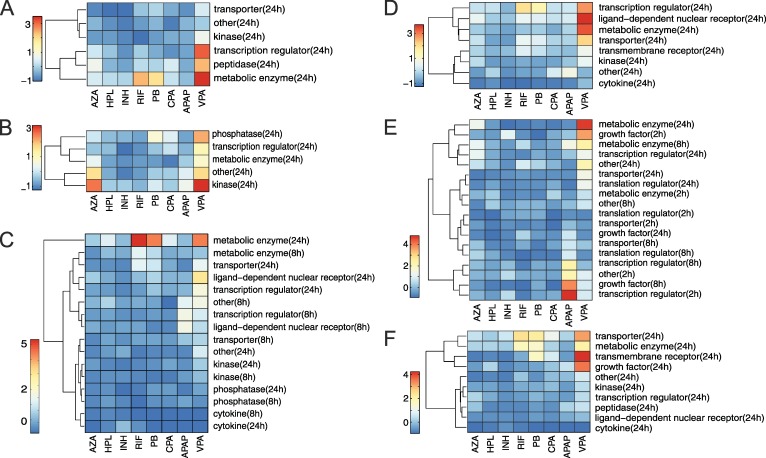
Toxic changes predicted for functional classes of genes involved in key cellular processes. The toxic changes were predicted for different functional classes of genes involved in the respective key cellular processes. All drugs belonging to the high-responsive group were considered. The color scale depicts toxic changes that were normalized over each heatmap. Normalization for each key cellular process is performed by subtracting the mean and by dividing the respective standard deviation.
A‘NRF-2 mediated oxidative stress response’.B‘Cell cycle G2/M DNA damage checkpoint regulation’.C‘PXR/RXR activation’.D‘LPS/IL-1 mediated inhibition of RXR function’.E‘Primary glomerulonephritis biomarker panel’.F‘Aryl hydrocarbon receptor signaling’.

RIF, PB and VPA demonstrated a high impact on metabolic enzymes involved in the NRF2-mediated oxidative stress response (Benjamini–Hochberg corrected p = 0.001, 95% CI = [0.11, 0.36], two-sample t-test), in particular on cytochrome P450 enzymes and transferases (Fig 7A). VPA further affected transcription regulators (Benjamini–Hochberg corrected p = 0.05, two-sample t-test, 95% CI = [0.06, 0.58]) particularly FOSL1 and KEAP1 (Fig 7A). A significant toxic change on kinases by AZA and VPA was observed at 24 h when focusing on processes of cell cycle G2/M DNA damage checkpoint regulation (Benjamini–Hochberg corrected p = 0.0002, 95% CI = [0.19, 0.45], two-sample t-test) (Fig 7B). A high toxic change of RIF, PB and VPA at 24 h was detected for metabolic enzymes involved in xenobiotic cytochrome P450 metabolism (Benjamini–Hochberg corrected p = 0.003, 95% CI = [0.38; 1.36], two-sample t-test) (S3A Fig), glutathione depletion induced by hepatocellular hypertrophy (Benjamini–Hochberg corrected p = 0.069, 95% CI = [0.05; 0.78], two-sample t-test) (S3D Fig), as well as in fatty acid metabolism (Benjamini–Hochberg corrected p = 0.0001, 95% CI = [0.12, 0.30], two-sample t-test) (S3E Fig), and in the activation of the PXR/RXR heterodimer (Benjamini–Hochberg corrected p = 0.001, 95% CI = [0.34, 1.02], two-sample t-test) (Fig 7C). Moreover, PB, VPA and RIF strongly perturbed BAX (Benjamini–Hochberg corrected p = 0.0016, 95% CI = [0.24, 0.47], two-sample t-test), an apoptosis regulator that modulates the mitochondrial permeability of the transporter VDAC [44] (Fig 7F). Investigating toxic changes of biomarkers referred to primary glomerulonephritis revealed a substantial impact of APAP on the heparin-binding growth factor HBEGF at 8 h (Benjamini–Hochberg corrected p = 0.0026, 95% CI = [0.42, 0.96], two-sample t-test) (Fig 7E).

Amongst others, a high impact of AZA and VPA on the regulation of the cell cycle G2/M DNA damage checkpoint was found in this second analysis (Fig 7B). Building on this observation, the cellular response on cell cycle regulation induced by both drugs was analyzed in more detail at the level of single genes and pathways in the following.

### Comparative toxicity analysis of azathioprine and valproic acid in cell cycle checkpoint regulation

**Fig 8:**
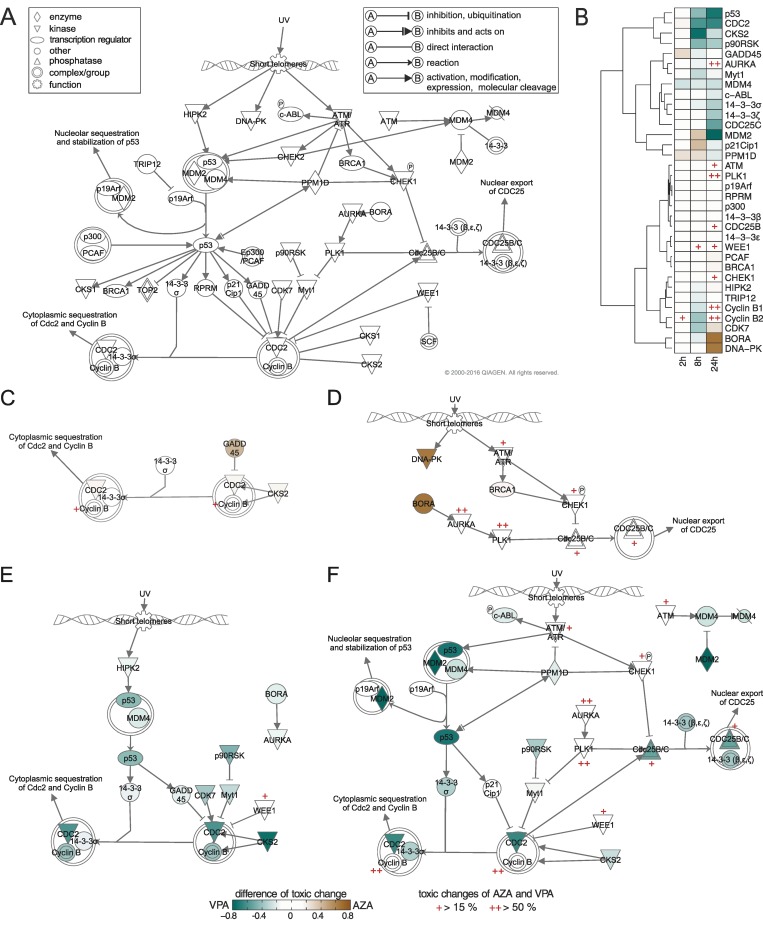
Comparison of toxic changes between AZA and VPA and differential response pathways at different timepoints. The color bar depicts differences of toxic changes between both drugs. Genes with high toxic changes for both drugs were explicitly marked in red.
APathway of ‘cell cycle G2/M DNA damage checkpoint regulation’.BComparison of toxic changes for involved genes between AZA and VPA.CAZA, 2 h.DAZA, 24 h.EVPA, 8 h.FVPA, 24 h.

The previous analysis of functional classes of genes revealed similar toxic behavior of AZA and VPA in the regulation of the cell cycle G2/M DNA damage checkpoint (Fig 7F) despite a significant pharmaceutical and chemical diversity (Table 1, S1 Table). We therefore considered the toxic behavior between AZA and VPA at the gene level in an exemplary use case by individually analyzing toxic changes of involved genes. The G2/M DNA damage checkpoint represents the second checkpoint in the cell cycle and ensures that genomic stability is maintained by repairing damaged DNA before entering the mitosis phase (Fig 8A) [45]. Hence, this pathway is crucially involved in DNA replication, recombination, and repair, respectively, and is consequently essential for cell viability [46]. A key role for the transition from the G2 phase to the M phase forms the cyclin-dependent kinases and several transcription regulators (Fig 8A) [47].

To directly compare the toxic behavior between both drugs, the differences of toxic change were calculated for all involved genes (Fig 8B). In this way, differentially responding genes of AZA and VPA reflected by a positive or negative value, respectively, could be identified. Analyzing differences in toxic changes revealed similar effects at 2 h for several genes (Fig 8B). Interestingly, only the p53 regulator MDM4, and the phosphatase PPM1D, the kinases CKS2 and CDC2 as well as the stress sensor GADD45, demonstrated high differences of toxic change for VPA and AZA, respectively (Fig 8B). Furthermore, a set of similarly responding genes was observed at 2 h, 8 h and 24 h (ATM, PLK1, p19Arf, RPRM, p300, 14-3-3(β,ε), CDC25B, WEE1 and CHEK1) (Fig 8B). Although these similarly responding genes showed only slight differences of toxic change, both drugs considerably affect ATM, CDC25B, WEE1 and CHEK1, and in particular PLK1 and cyclin B1 and B2 (Fig 8B). In contrast to the findings at 2 h, differences in several genes were found at 8 h and 24 h such as CKS2, CDC2, and p53 for VPA and p21Cip1, DNA-PK and BORA for AZA (Fig 8B).

The differentially responding genes were next used to build differential response pathways at given timepoints (Fig 8C–8F). Note that none of these pathways could be found for AZA and VPA alone at 2 h and 8 h, respectively, but can only be identified through a comparative analysis. Exploring these pathways helps to compare dynamic changes between AZA and VPA in the regulation of the cell cycle G2/M DNA damage checkpoint when switching from therapeutic to toxic dose administration.

Analyzing differential response pathways of AZA at 2 h (Fig 8C) and VPA at 8 h (Fig 8E) revealed that AZA highly perturbed GADD45 and CKS2, which regulates CDC2-cyclin B complex, while VPA affected the same key complex by strongly perturbing p53 via HIPK2, on the one hand, and p90RSK, Myt1, and CDK7, on the other hand. Interestingly, GADD45 and CKS2 were involved in both pathways but in a time-shifted manner. In order to regulate key processes of the cell cycle G2/M checkpoint at 24 h, BORA and DNA-PK were highly affected by AZA (Fig 8D). In contrast, a significantly higher activity due to VPA administration was observed at the same timepoint thereby regulating all major processes mostly via p53, MDM2 and CDC2 (Fig 8F).

The comparative analysis of similarly- and differentially responding genes might help to identify either individually or commonly affected molecular biomarkers that reflect toxic drug action, which is either exclusively induced by a single drug (e.g., BORA at 24 h for AZA) or simultaneously by both drugs (e.g., cyclin B1 and B2 at 24 h). Genes that are simultaneously affected by two drugs might also be a common target during drug co-administration as such leading to an additive drug effect.

### Analysis of individual genes

To conclude our analysis, toxic changes were calculated for individual genes that were involved in the key cellular processes strongly affected by the high-responsive drugs (Fig 7, S3 Fig). These gene-related toxic changes were then used to quantitatively explore which genes were similarly perturbed by which drugs. This knowledge was finally used to identify individual and common molecular biomarkers for single drugs and subset of drugs, respectively.

Molecular biomarkers play a key role in clinical risk assessment and the early prediction of drug toxicity. To identify robust common molecular biomarkers within the cluster of high-responsive drugs, a significant and similar toxic change (at least one and a half-fold increase and less than half of the standard deviation) at a certain timepoint was required. To test whether the common molecular biomarkers were sensitive, the respective toxic changes of an identified biomarker were compared between the low- and high-responsive drugs.

In total, twelve common molecular biomarkers were detected for the set of high-responsive drugs (Table 2). Nine genes demonstrated statistical significant changes (Benjamini–Hochberg corrected p < 0.05): the metabolizing enzymes EPHX1, CYP2C9, SULT1A2, and GSTP1, the transporter ABCA1, as well as the kinases PRKACA and MAP3K14, and the ligand-dependent nuclear receptors AHR and NR0B2 (Table 2). These biomarkers are involved in key cellular processes such as in the activation of the PXR/RXR heterodimer, in the LPS and IL-1 mediated inhibition of the RXR function, or in the aryl hydrocarbon receptor signaling (Table 2). In contrast, the transcription regulator ELF3, the growth factor TGFB2 and the kinase PKMYT1 were not found to be significant (Benjamini–Hochberg corrected p ≥ 0.05, two-sample t-test) indicating that these genes show similar toxic change for both the high- and the low-responsive drugs (Table 2).

**Table 2:** Common molecular biomarkers. Common molecular biomarkers were identified in different key cellular processes at different timepoints for the drugs of the high-responsive group. Benjamini-Hochberg corrected p-values p were calculated by comparing the correspondent toxic changes between the low and high-responsive group. Functional types were taken from QIAGENs Ingenuity Pathway Analysis (IPA, QIAGEN Redwood City, www.qiagen.com/ingenuity).

Gene	Functional type	p-value	Key cellular processes (timepoint)
EPHX1	peptidase	0.001*	- xenobiotic metabolism signaling (24 h)
			- NRF-2 mediated oxidative stress response (24 h)
CYP2C9	enzyme	0.003*	- cytochrome p450-substrate is a xenobiotic (8 h)
			- PXR/RXR activation (8 h)
			- CAR/RXR activation (8 h)
ABCA1	transporter	0.003*	- LPS/IL-1mediated inhibition of RXR function (24 h)
GSTP1	enzyme	0.004*	- xenobiotic metabolism signaling (24 h)
			- aryl hydrocarbon receptor signaling (24 h)
			- LPS/IL-1 mediated inhibition of RXR function (24 h)
			- NRF-2 mediated oxidative stress response (24 h)
SULT1A2	enzyme	0.004*	- xenobiotic metabolism signaling (24 h)
			- LPS/IL-1mediated inhibition of RXR function (24 h)
AHR	ligand-dependent nuclear receptor	0.005*	- xenobiotic metabolism signaling (24 h)
			- aryl hydrocarbon receptor signaling (24 h)
PRKACA	kinase	0.008*	- PXR/RXR activation (24 h)
MAP3K14	kinase	0.016*	- xenobiotic metabolism signaling (24 h)
NR0B2	ligand-dependent nuclear receptor	0.022*	- PXR/RXR activation (24 h)
			- aryl hydrocarbon receptor signaling (24 h)
			- LPS/IL-1 mediated inhibition of RXR function (24 h)
TGFB2	growth factor	0.086	- aryl hydrocarbon receptor signaling (24 h)
PKMYT1	kinase	0.186	- cell cycle G2/M DNA damage checkpoint regulation (24 h)
ELF3	transcription regulator	0.212	- primary glomerulonephritis biomarker panel (8 h)

* Benjamini–Hochberg corrected p < 0.05

**Fig 9:**
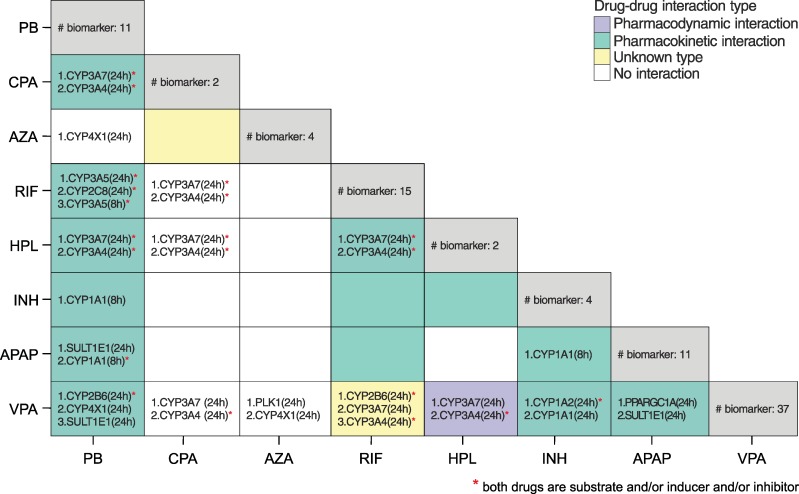
Potential DDIs between the high-responsive drugs. The total number of identified biomarkers for each drug is shown on the diagonal. The biomarkers were ranked according to the absolute differences of toxic change between both considered drugs. Measures of the performance were additionally calculated by comparing predicted DDIs with known DDIs from literature: accuracy = 68%, sensitivity = 75%, specificity = 58%, and precision = 71%.

To identify individual molecular biomarkers for each of the high-responsive drugs, a very strong toxic change (at least seven-fold increase compared to mean toxic change) was required. The majority of the individual molecular biomarkers belong to the cytochrome P450 family, transcription regulators, or they are transporters (S3 Dataset). These drug-specific molecular biomarkers were finally analyzed to identify potential DDIs between the high-responsive drugs in the case of co-administration. To this end, a potential DDI between two drugs was assumed, if both drugs share at least one biomarker (S4 Dataset). The consequently identified pairs of drugs were then compared with known DDIs from DrugBank [23] and from Drugs.com (Fig 9). Strikingly, the prediction of DDIs reaches an accuracy of 68% and a precision of 71% with respect to DDIs known from the literature (Fig 9). The number of correctly predicted DDIs and non-DDIs was found to be 75% and 58%, respectively. Analyzing all potential DDIs, 35 out of the 42 DDIs were identified based on high toxic changes on cytochrome P450 enzymes for both drugs (S4 Dataset). Interestingly, in 72% of these cases predicted cytochrome P450 enzymes are in accordance with literature data [23] supporting the potential validity of the approach.

## Discussion

In this article, a comparative study of drug-induced hepatotoxicity was presented, which enables the investigation and evaluation of the hepatotoxic potential of several drugs within a patient context. Toxic changes reflecting time-resolved cellular responses induced by oral drug administration of therapeutic and toxic doses in humans were thereby predicted to study changes in key cellular processes, functional classes of genes, and individual genes, as well as to identify molecular biomarkers and potential DDIs. Notably, toxic changes describe the transition from therapeutic drug response to adverse events and thus allow a quantitative representation of clinically relevant situations within a patient context.

By applying PICD (Fig 1) [20], in vitro toxicity data obtained in primary human hepatocytes from Open TG-GATEs [21] could be contextualized to predict in vivo drug response patterns of key cellular processes for the simulated therapeutic and toxic PK profiles (Fig 4, Fig 6). As input for PICD, drug-specific human PBPK models were developed and validated with different dosage regimens used in previous clinical studies (Fig 3, S3 Table). This validation step ensures reliable predictions of PK profiles for a wide range of in vivo doses since potential non-linearity’s are explicitly taken into account. Therapeutic and toxic drug concentrations over time were then simulated (Fig 4). The toxic doses were identified from clinical cases for which toxic events occurred (S8 Table). Two large databases as well as literature were screened to reasonably cover a wide range of toxic doses (S8 Table). Moreover, it should be noted that the in vivo doses considered here reflect the range of drug exposure occurring in clinical practice (S3 Table, S8 Table).

When evaluating the toxic behavior between the fifteen hepatotoxic drugs, no significant toxic change was observed in the case of SST, AD or DFN (Fig 6). However, it is known that these drugs may still have a high hepatotoxic potential [48,49,50]. Notably, these three drugs are highly bound to plasma proteins in vivo and are rapidly metabolized such that high in vivo doses are necessary to reach the in vitro exposure when applying PICD [20]. The identified toxic in vivo doses are therefore probably higher than those provided in the literature (S8 Table). As a consequence, the drug responses predicted by PICD for the toxic doses may be very low. Hence, a future application of drug-specific pharmacokinetics in in vitro assay design might improve the in vivo relevance of certain in vitro outcomes. To validate predicted drug response profiles of all considered drugs, in vivo rat data from Open TG-GATEs were used [21]. In a preparatory proof-of-concept analysis, correlation results in rats demonstrated that PICD-based predictions were generally in concordance with in vivo observations (Fig 5). Although uncertainties were observed in some cases, it can still be assumed that the predicted drug responses in humans have in vivo relevance since such uncertainties are almost unavoidable due to (i) the high variability of physicochemical properties and pharmacological diversity of the considered drugs, (ii) the several differences potentially influencing the response data observed in vitro and in vivo (e.g. different plasma protein binding and enzyme and transporter activity, crosstalk between relevant tissues and organs in the in vivo situation), (iii) the time-dependent interpolation that was necessary to make the predictions comparable to the in vivo observations [21].

In the first analysis of the comparative toxicity study, toxic changes in significantly perturbed key cellular processes were compared between the fifteen hepatotoxic drugs (Fig 6). One objective of this study was to investigate whether subsets of drugs exist, which share similar perturbations of key cellular processes, and whether these subsets have common pharmacokinetic parameters or drug-specific characteristics such as DILI-potential, solubility and permeability properties, and the target organ or system, respectively. Surprisingly the analyses showed that the low-responsive drugs primarily belong to the BCS class 2 (high permeability, low solubility), except PHE (class 1: high permeability, high solubility). In contrast, high-responsive drugs were rather less permeable with statistically significant differences for lipophilicity but not for water solubility. This finding might imply that a low permeability plays an important role in the hepatotoxic potential of the considered drugs in contrast to the results of other studies that showed a correlation between high lipophilicity and toxicological outcomes [51,52]. This could be due to the fact that in our multiscale approach additional drug properties such as plasma protein binding or doses applied in vivo are implicitly taken into account in the whole-body PBPK models and set in relation to actual in vitro omics data for known hepatotoxicants. Also, it could be hypothesized that hydrophilic drugs tend to have more polar functional groups and thus are more prone to enzyme-mediated adverse chemical modifications since these drugs present several potential interaction targets within the cell. Statistically significant differences between both groups were identified for the plasma protein binding but not for the ratio of toxic and therapeutic area under the curves and dose levels, respectively. The latter result is important, since it demonstrates that the hepatotoxic potential is not affected by the selection of the therapeutic and toxic dose levels and the resulting concentration-time courses. Interestingly, the low-responsive drugs tend to have a narrow therapeutic index (defined as the ratio between toxic and therapeutic dose) (Fig 4, S8 Table) [53], which increases the risk of adverse reactions following high drug exposure due to overdosing or idiosyncrasy.

Next, the toxic changes between the high-responsive drugs were predicted in terms of functionally-related genes involved in key cellular processes. In this way, toxic changes of functional classes (e.g., phosphatases or transcription regulators) that are mainly contributing to a certain key cellular process could be identified. For instance, a high toxic change of growth factors at 8 h was found for APAP, which highly increases the risk of renal impairment as described in previous studies [54,55] (Fig 7E). In the case of AZA and VPA, a high toxic change in kinases was found at 24 h, which were involved in the regulation of the cell cycle G2/M DNA damage checkpoint (Fig 7B). This is in striking accordance with previous studies [56,57,58,59] where both drugs were also reported to have a substantial impact on the cell cycle regulation. The hepatotoxic potential of AZA and VPA in this crucial pathway was hence exemplarily investigated in more detail to compare toxic changes of involved genes. Focusing on the cyclins B1 and B2 or the kinase PLK1, for instance, revealed similar toxic changes and especially high drug responses at 24 h for both drugs. This suggests a potential key role of these genes in the drug-induced hepatotoxicity of AZA and VPA. In contrast, differentially responding genes for both drugs could be found at different timepoints (Fig 8B). Interestingly, AZA and VPA similarly perturbed central biological processes of the G2/M DNA damage checkpoint (Fig 8). However, the initiation of these processes is complementary and preferably occurred by DNA-PK, GADD45 and BORA for AZA, and CDC2, p53 and MDM2 for VPA (Fig 8).

Finally, the calculated toxic changes were used to discover common and individual molecular biomarkers for the high-responsive drugs. A set of nine common molecular biomarkers could be identified, which showed significant differences to the low-responsive drugs indicating a high sensitivity of the identified biomarkers (Table 2). Moreover, individual molecular biomarkers mostly enzymes of the cytochrome P450 family, were found and further used to detect potential DDIs. Here, the identification of potential DDIs was based on high toxic changes reflecting differences between therapeutic and toxic drug response. Known DDIs from literature could be predicted with a precision of 71% (Fig 9). In some cases, known DDIs from literature (e.g., between RIF and INH) were not identified as such, (Fig 9), which might indicate that these interactions are only significant after therapeutic drug administration. In contrast, predicted DDIs not found in literature might present newly discovered drug interactions, which only occur under toxic conditions. The consideration of more toxic and non-toxic drugs in a future extension of our analysis could further improve the identification and validation of molecular biomarkers and DDIs discovered in an in vivo situation. Moreover, it is also conceivable to apply the workflow on a set of candidate drugs during early drug development. In this regard, measured time-series gene expression profiles could be contextualized in human PBPK models parametrized based on molecular modeling to identify potential toxic and non-toxic compounds before entering the clinical phases.

To conclude, the hepatotoxic potential of a set of known hepatotoxic drugs was studied and compared by predicting toxic changes for humans, which reflect the transition from therapeutic drug response to toxic reactions. We therefore analyzed primary human hepatocytes at the cellular level, and developed human PBPK models at the organism level and coupled both levels by the application of the recently developed approach called PICD (Fig 1). Hence, the analysis of toxic changes allows a quantitative evaluation of clinically relevant situations within a patient context. Altogether, toxic changes after 2 h, 8 h and 24 h in significantly affected key cellular processes could be analyzed thereby identifying a low-responsive (SST, DFN, AD, ERY, FT, CSA and PHE) and a high-responsive group (RIF, CPA, PB, INH, HPL, AZA, APAP and VPA) (Fig 6). For the latter, molecular biomarkers and potential DDIs could be identified. An accuracy, specificity, sensitivity, and precision of 67%, 58%, 75%, and 71%, respectively, has been reached when comparing the potential DDIs with known DDIs from literature. Notably, 72% of the predicted cytochrome P450 enzymes could be identified in known drug-enzyme association for both drugs involved in the specific DDI [23]. This article provides a systematic analysis of drug-induced hepatotoxicity by coupling in vitro toxicity data measured in primary human hepatocytes [21] with in vivo pharmacokinetics, and thus allows an investigation of differences in drug response following oral administration of therapeutic and toxic doses in humans. Drug-induced hepatotoxicity could be hence analyzed within a patient context to investigate drug effects between therapeutic and toxic conditions and to discover molecular biomarkers as well as potential DDIs for several hepatotoxic drugs. The results of our study might help to improve clinical risk assessment and patient safety during a drug development process in the future.

## Materials and Methods

### Development of whole-body PBPK models

The whole-body PBPK models of the fifteen considered drugs (S1 Text) were built by use of the software PK-Sim (version 6.0) and MoBi (version 3.4) (Bayer Technology Services, GmbH, Leverkusen, Germany) [60,61], which are freely available for academic use. PBPK models describe ADME processes based on prior information about the physicochemical properties of a drug and the physiology and anatomy of the organism [61]. In the PBPK model structure, relevant tissues and organs are represented by compartments and are connected by blood flow (S4 Fig). These compartments are usually subdivided into plasma, red blood cells, interstitial and intracellular space. Distribution models describing mass transfer are parameterized based on physicochemical drug properties and are used to determine partition coefficients as well as cellular permeability values between these compartments [62,63,64,65]. The best-performing calculation methods provided in the modeling software were used in the developed PBPK models (S7 Table).

A reference PBPK model for intravenous administration was first developed and assessed by comparing simulated drug concentrations with experimental data from literature. For flutamide, only a reference PBPK model for oral administration of 250 mg was developed, since this is the major therapeutic dose level and administration route. The Michaelis-Menten constant (Km) and the maximal velocity (vmax) representing the kinetic behavior of active processes were mainly fitted to best describe the experimental data used for model establishment. However, experimentally measured Km values for several metabolic reactions could be identified in literature and were used unchanged in the model structure (S4 Table). In the PBPK model of INH, two different vmax values were estimated for the enzymatic reaction catalyzed by NAT2 to characterize fast and slow metabolizer, for which clinical data were available [66,67]. Note that NAT2 polymorphism may extensively influence the pharmacokinetic and pharmacodynamic behavior of INH for specific patient subgroups. To describe the elimination of the drugs and their metabolites, renal and biliary clearance processes were incorporated into the PBPK models (S5 Table) In the case of AZA, 6-MP, AD and CSA, renal elimination was not considered since negligible amounts were found in urine [23,68].

Once a sufficient model quality was reached, a reference PBPK model for oral administration was developed thereby using all parameters identified for the intravenous reference PBPK model. Only the intestinal permeability was adjusted in some cases to best describe the absorption phase after oral drug intake (S6 Table). In general, an endothelial barrier between the plasma and the interstitial space is assumed for large molecules like proteins but not for small molecules [69]. In the PBPK model of DFN, however, the rate of permeation through this endothelial barrier was limited in all organs except in the liver (brain: 0.004 cm/s, other organs/tissues: 0.04 cm/s), since DFN is highly bound to plasma proteins (Table 1).

The established reference PBPK models for both administration routes were further validated dependent on the availability of experimental data from other clinical studies. Since APAP and SST are mostly administered orally, only one administration route was considered in the specific PBPK models (Fig 2). In the case of ERY, erythromycin ethylsuccinate (ERY-PED) [70], an ester of the base form, was orally administered. In the validation step, all parameters of the specific reference PBPK model were left unchanged, except parameters characterizing the specific individuals and the dosage regimen. In the validated PBPK model established for intravenous and oral administration of 200 mg and 300 mg of CPA, respectively, (Fig 2, S3 Table), kidney plasma clearance was reduced to 5.1 ml/h/kg for renally-impaired patients [71,72].

Finally, a normalized root-mean-square deviation (RMSD) as well as the coefficient of determination (R^2^) identified after linear regression were calculated for all human PBPK models to assess the model quality [20].

To develop rat PBPK models used for the validation cross-species extrapolation was applied. Thereby, pharmacokinetics were extrapolated from humans to rats by taking into account physiological and anatomical differences between both species [42].

### Prediction and validation of in vivo drug responses

The integrative multiscale approach called PICD allows a time-resolved description of drug-induced in vivo response at the patient level by integrating in vitro toxicity data (S1 Text) into whole-body PBPK models [20]. Here, PICD was applied on the fifteen hepatotoxic drugs to predict in vivo drug responses of key cellular processes, functional classes of genes, and individual genes, induced by oral administration of therapeutic and toxic doses in humans (S1 Text). When applying PICD, bioavailability values calculated from the developed human PBPK model were used to consider oral administration (S9 Table). In the case of AZA, the bioavailability found in literature was used since the difference between the literature value and the calculated value was significantly high [73]. In vivo drug responses after 2 h, 8 h and 24 h for therapeutic and toxic dose administration (S1 Text) were then calculated by computing the mean gene response level (gene response is defined as absolute log2 fold change) of all genes assigned to a specific key cellular process (S2 Table). In the case where in vitro data [21] only exist for 8 h and 24 h, the predicted response patterns were interpolated to determine response values at 2 h. When analyzing functional classes of genes, in vivo drug responses were predicted for the different functional classes of genes involved in a specific key cellular process by calculating the mean gene response level of all genes assigned to a certain functional category.

To validate PICD in rats, significantly enriched key cellular processes (Benjamini-Hochberg corrected p < 0.01) were first identified for each drug (S1 Dataset) and correspondent in vivo drug responses were then predicted following oral administration of the three doses applied in the in vivo rat study [21]. Here, the highest dose was identified in a 4-week toxicity study [21]. According to [20], predictions were subsequently compared to in vivo observations by calculating the Pearson correlation coefficient r, the coefficient of determination R^2^ and corresponding confidence intervals (CI) between predicted drug response profiles and measurements obtained in rat livers [21]. Predicted drug response profiles were linearly interpolated to be comparable to time-matched in vivo measurements (3 h, 6 h, 9 h, and 24 h).

### Identification of significantly perturbed key cellular processes

In the first analysis of the comparative toxicity study, a set of strongly perturbed key cellular processes was extracted by considering all processes that were found to be significantly overrepresented (Benjamini-Hochberg corrected p < 0.01) in the in vitro experiment [21] by at least one third of the hepatotoxic drugs, irrespectively of the timepoints (S2 Dataset). In the second analysis, a toxic change of at least 10%, on average, was required to identify a set of key cellular processes significantly affected at certain timepoints by the high-responsive drugs but not by the low-responsive drugs. At this threshold, no key cellular process was perturbed at any timepoint by the low-responsive drugs.

### Calculation of toxic changes

In the comparative toxicity analysis (Fig 2), toxic changes were calculated at different timepoints (2 h, 8 h and 24 h) for key cellular processes, functional classes of genes within a key cellular process, and single genes. Here, a toxic change at a timepoint t for a drug d is defined as follows:

$${toxic\;change}_{t,x,d}={in\;vivo\;drug\;response\;(toxic)\;}_{t,x,d}-{in\;vivo\;drug\;response\;(therapeutic)}_{t,x,d}$$ (1)

where x denotes a key cellular process, a functional class within a key cellular process, or a single gene. In vivo drug responses induced by therapeutic and toxic dose administration were predicted by calculating gene response levels (defined as absolute log2 fold change) for single genes, and by calculating the mean gene response level of all genes assigned to a key cellular process or to a functional class within a key cellular process, respectively [20]. In order to compare the toxic behavior of AZA and VPA in cell cycle checkpoint regulation, differences of toxic changes for all involved genes were calculated between both drugs and were mapped onto the pre-defined pathway ‘cell cycle G2/M DNA damage checkpoint regulation’ taken from QIAGEN’s Ingenuity Pathway Analysis (IPA, QIAGEN Redwood City, www.qiagen.com/ingenuity). Note that differentially responding genes (absolute difference of toxic change > 0.15) of AZA and VPA are reflected by a positive and a negative value, respectively. All differentially responding genes as well as genes with toxic changes higher than 15% for both drugs were finally used to build differential response pathways.

### Prediction of molecular biomarkers and potential DDIs

All genes involved in the strongly affected key cellular processes analyzed in the functional analysis were considered to identify potential molecular biomarkers and DDIs. A gene g was marked as common molecular biomarker for all high-responsive drugs if the following condition was fulfilled:

$$mean{\left(toxic\;change\right)}_{g}>\;1.5*mean(toxic\;change)\;AND\;std{\left(toxic\;change\right)}_{g}<\;0.5*std(toxic\;change)$$ (2)

In contrast, a gene g was marked as individual molecular biomarker for only a single drug d if the more stringent requirement was fulfilled:

$${\left(toxic\;change\right)}_{d,g}>\;7*mean(toxic\;change)$$ (3)

Several thresholds deviating from ±5% of the used thresholds above did not significantly alter the number of identified common molecular biomarkers (±14%) or individual molecular biomarkers (±4%). All common molecular biomarkers were additionally compared between the low and high-responsive drugs by evaluating the correspondent toxic changes between both groups. All individual molecular biomarkers were used to identify potential DDIs. Thereby, a potential DDI was assumed, if at least one individual molecular biomarker was identified for both drugs. These DDIs were then compared with known DDIs from DrugBank [23] and from Drugs.com [Accessed 2016 March 3rd] by calculating the accuracy, sensitivity, specificity and the precision that were formulated as follows:

$$Accuracy=\;\frac{TP+TN}{TP+FP+TN+FN}$$ (4)

$$Sensitivity=\;\frac{TP}{TP+FN}$$ (5)

$$Specificity=\;\frac{TN}{TN+FP}$$ (6)

$$Precision=\;\frac{TP}{TP+FP}$$ (7)

where TP represents true positive, TN represents true negative, FP represents false positive, FN represents false negative.

Types of DDIs (‘pharmacokinetic interaction’ and ‘pharmacodynamic interaction’) were assigned according to [74], if the interaction type was not unknown. The BioInteractor tool from DrugBank was used to confirm predicted drug-enzyme associations for two corresponding drugs involved in a potential DDI [23].

## Supporting Information

S1 Fig PBPK model assessment. Simulated concentration-time profiles of parent drugs and their metabolites were compared to experimental PK data. Observed vs. predicted plots including the RMSD value and the coefficient of determination (R^2^) were generated for all reference and validated PBPK models. All p-values calculated for the R^2^ values were lower than 0.0001.
(PDF)

S2 Fig Principal component analysis. Principal component analysis was applied on all toxic changes predicted at 2 h (blue), 8 h (green), and 24 h (red). Percentage of explained variance of principal components one (PC1) and two (PC2) are shown in brackets. Ellipses around the different groups are generated with a confidence level of 0.95. Results of principal component analysis were visualized by use of the web tool ClustVis [76].
(PDF)

S3 Fig Toxic changes predicted for functional classes of genes involved in key cellular processes. The toxic changes were predicted for different functional classes of genes involved in the respective key cellular processes. All drugs belonging to the high-responsive group were considered. The color scale depicts toxic changes that were normalized over each heatmap. Normalization for each key cellular process is performed by subtracting the mean and by dividing the respective standard deviation.
A‘Cytochrome P450 –substrate is a xenobiotic’.B‘CAR/RXR activation’.C‘Xenobiotic metabolism signaling’.D‘Glutathione depletion–hepatocellular hypertrophy’.E‘Fatty acid metabolism’.
(PDF)

S4 Fig Schematic representation of a multiscale whole-body PBPK model. Schematic representation of a multiscale whole-body PBPK model including 15 different tissues and organs that are connected by blood flow. Sub-compartmentalization into blood cells, blood plasma, interstitial and intracellular space is exemplarily presented for a default compartment. (Reproduced with permission [20], https://creativecommons.org/licenses/by/4.0/)
(PDF)

S1 Table Drug-specific annotations. DILI-potential, severity score, anatomical main group, therapeutic and chemical subgroup as well as BCS class of the fifteen considered drugs.
(DOCX)

S2 Table Toxicity lists. Seventy-four toxicity lists representing key cellular processes were taken from QIAGENs Ingenuity Pathway Analysis (IPA, QIAGEN Redwood City, www.qiagen.com/ingenuity).
(DOCX)

S3 Table Experimental conditions. Administration route (intravenous (iv), or oral (po)), respective doses, number of subjects and health state. The experimental PK data were either used for establishment of the reference PBPK model (Reference) or for model validation (Validation).
(DOCX)

S4 Table Active drug transport and metabolic processes. Metabolic and active drug transport processes either consist of the metabolic enzyme and the corresponding metabolite or of the transporter and the corresponding transporter type (efflux, influx). Kinetic parameters Km and vmax were used to characterize the kinetic behavior of active processes. A liver plasma clearance of 11.5 ml/min/kg was estimated for the clearance of 2-hydroxy-FT. For INH, NAT2 polymorphism was considered by estimating two different vmax values to best describe clinical data available for fast and slow metabolizer [66,67].
(DOCX)

S5 Table Renal and biliary clearance processes. Renal and biliary clearance processes of the developed PBPK models.
(DOCX)

S6 Table Intestinal permeability values. Intestinal permeability values for all drugs and their metabolites. Some intestinal permeability values originally provided by the modeling software [60] (Initial intestinal permeability) were slightly adjusted (Intestinal permeability used in model) to best describe the experimental data for oral administration.
(DOCX)

S7 Table Calculation methods for partition coefficients and cellular permeability values. Different calculation methods used in the established PBPK models to calculate intracellular to plasma partition coefficients as well as permeability values between interstitial and cellular space. The calculation methods are provided in the modeling software [60].
(DOCX)

S8 Table Toxic dose levels. Toxic dose levels for the fifteen drugs were identified by database and literature screening. To determine a toxic dose for SST and FT, toxic rat doses [21] were scaled since no appropriate doses were found in literature.
(DOCX)

S9 Table Bioavailability values. Bioavailability values after 24 h calculated by use of the modeling software PK-Sim [60]
(DOCX)

S1 Dataset Over-representation analysis for rats. Significantly overrepresented key cellular processes identified in rat hepatocytes.
(XLS)

S2 Dataset Over-representation analysis for humans. Significantly overrepresented key cellular processes identified in human hepatocytes.
(XLS)

S3 Dataset Molecular biomarkers. Individual molecular biomarkers identified for the high- and low-responsive drugs.
(XLSX)

S4 Dataset Drug-drug interactions. Drug-drug interactions predicted for the high-responsive drugs.
(XLSX)

S1 Text Supplementary Materials & Methods. (DOCX)

S1 Model Files Supplementary model files for PBPK simulations. (ZIP)
