# Supplementary figures and images for: A Comparative Analysis of Drug-Induced Hepatotoxicity in Clinically Relevant Situations
Source: PLoS Comput Biol. 2017 Feb 2;13(2):e1005280. doi: 10.1371/journal.pcbi.1005280 (PMC5289425; doi:10.1371/journal.pcbi.1005280)

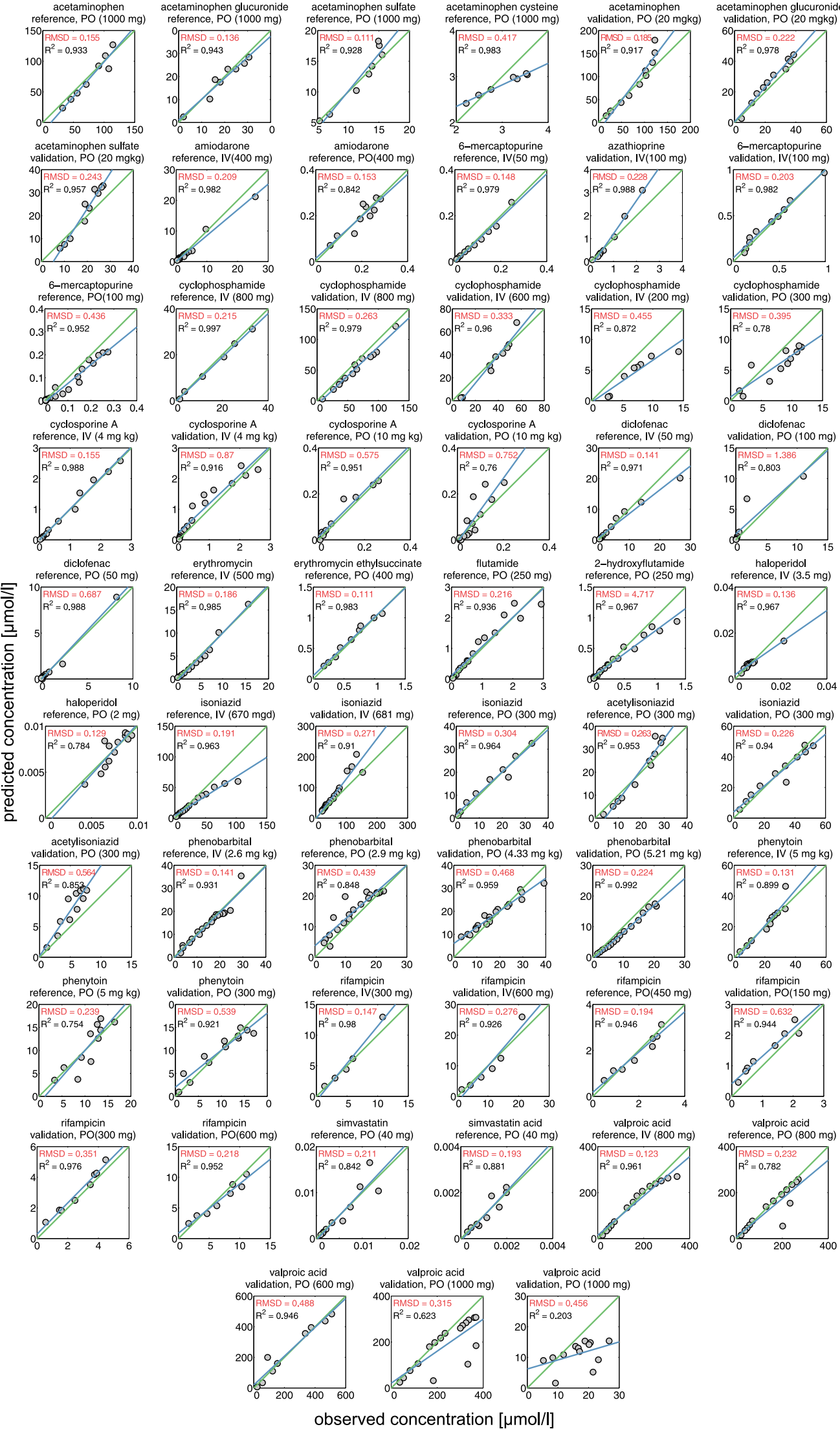

Supplement: S1 Fig — Simulated concentration-time profiles of parent drugs and their metabolites were compared to experimental PK data. Observed vs. predicted plots including the RMSD value and the coefficient of determination (R2) were generated for all reference and validated PBPK models. All p-values calculated for the R2 values were lower than 0.0001. (PDF) [file pcbi.1005280.s001.pdf]

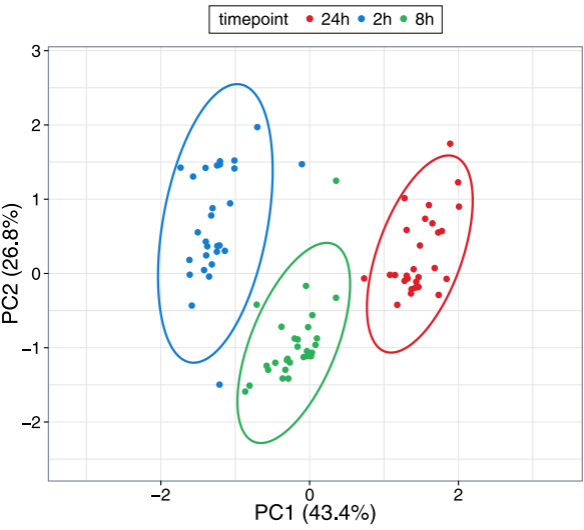

Supplement: S2 Fig — Principal component analysis was applied on all toxic changes predicted at 2 h (blue), 8 h (green), and 24 h (red). Percentage of explained variance of principal components one (PC1) and two (PC2) are shown in brackets. Ellipses around the different groups are generated with a confidence level of 0.95. Results of principal component analysis were visualized by use of the web tool ClustVis [76]. (PDF) [file pcbi.1005280.s002.pdf]

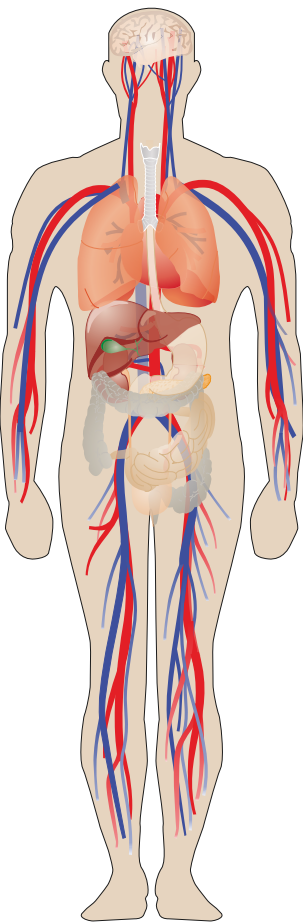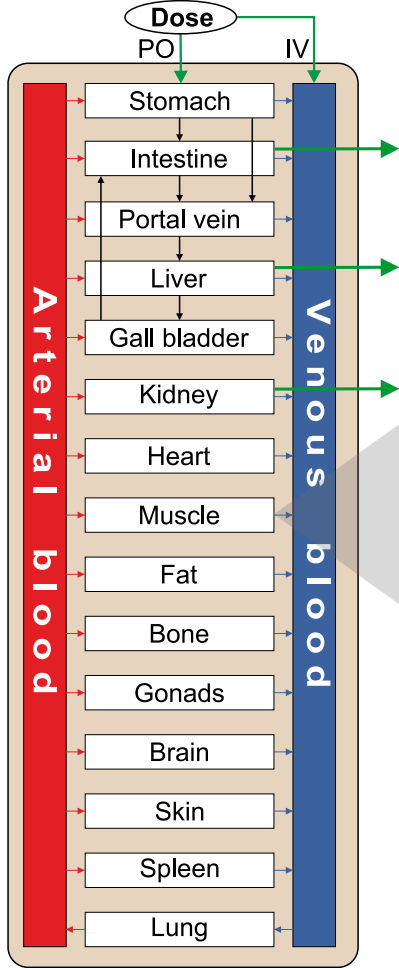

#### Organ/Tissue

Blood cells

Plasma

Interstitium

Intracellular

Supplement: S4 Fig — Schematic representation of a multiscale whole-body PBPK model including 15 different tissues and organs that are connected by blood flow. Sub-compartmentalization into blood cells, blood plasma, interstitial and intracellular space is exemplarily presented for a default compartment. (Reproduced with permission [20], https://creativecommons.org/licenses/by/4.0/) (PDF) [file pcbi.1005280.s004.pdf]
