# Supplementary material for: A Comparative Analysis of Drug-Induced Hepatotoxicity in Clinically Relevant Situations
Source: PLoS Comput Biol. 2017 Feb 2;13(2):e1005280. doi: 10.1371/journal.pcbi.1005280 (PMC5289425; doi:10.1371/journal.pcbi.1005280)
Supplement: S3 Fig — The toxic changes were predicted for different functional classes of genes involved in the respective key cellular processes. All drugs belonging to the high-responsive group were considered. The color scale depicts toxic changes that were normalized over each heatmap. Normalization for each key cellular process is performed by subtracting the mean and by dividing the respective standard deviation. A‘Cytochrome P450 –substrate is a xenobiotic’.B‘CAR/RXR activation’.C‘Xenobiotic metabolism signaling’.D‘Glutathione depletion–hepatocellular hypertrophy’.E‘Fatty acid metabolism’. (PDF) [file pcbi.1005280.s003.pdf]

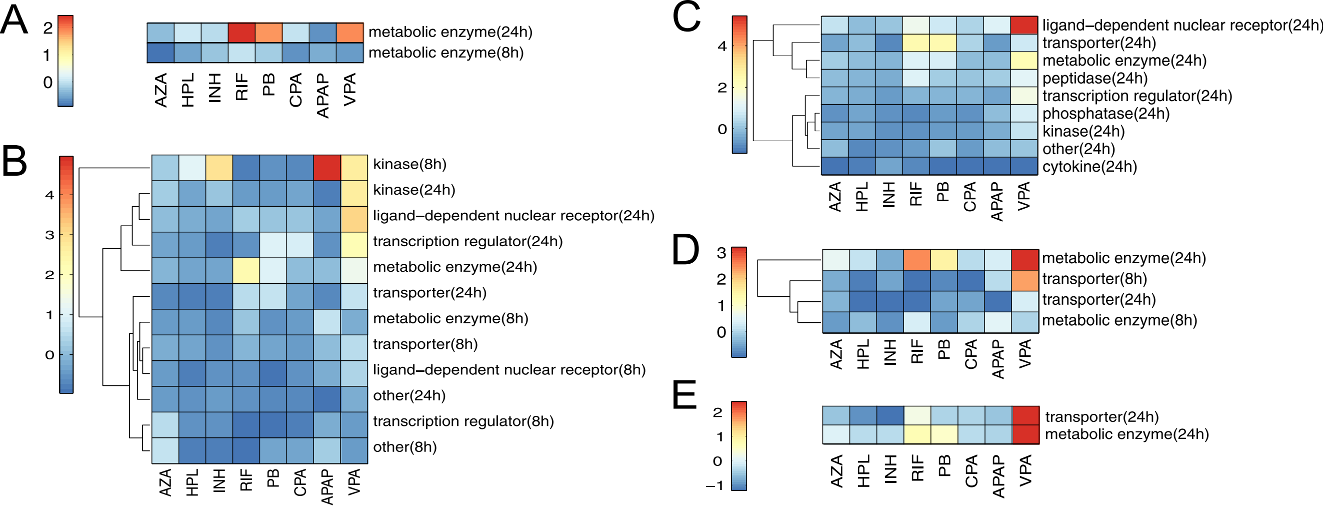

A
2
1
0
metabolic enzyme(24h)
metabolic enzyme(8h)
AZA
HPL
INH
RIF
PB
CPA
APAP
VPA
B
4
3
2
1
0
kinase(8h)
kinase(24h)
ligand−dependent nuclear receptor(24h)
transcription regulator(24h)
metabolic enzyme(24h)
transporter(24h)
metabolic enzyme(8h)
transporter(8h)
ligand−dependent nuclear receptor(8h)
other(24h)
transcription regulator(8h)
other(8h)
AZA
HPL
INH
RIF
PB
CPA
APAP
VPA
C
4
2
0
ligand−dependent nuclear receptor(24h)
transporter(24h)
metabolic enzyme(24h)
peptidase(24h)
transcription regulator(24h)
phosphatase(24h)
kinase(24h)
other(24h)
cytokine(24h)
AZA
HPL
INH
RIF
PB
CPA
APAP
VPA
D
3
2
1
0
metabolic enzyme(24h)
transporter(8h)
transporter(24h)
metabolic enzyme(8h)
AZA
HPL
INH
RIF
PB
CPA
APAP
VPA
E
2
1
0
−1
transporter(24h)
metabolic enzyme(24h)
AZA
HPL
INH
RIF
PB
CPA
APAP
VPA
