# Supplementary material for: A Comparative Analysis of Drug-Induced Hepatotoxicity in Clinically Relevant Situations
Source: PLoS Comput Biol. 2017 Feb 2;13(2):e1005280. doi: 10.1371/journal.pcbi.1005280 (PMC5289425; doi:10.1371/journal.pcbi.1005280)
Supplement: S1 Table — DILI-potential, severity score, anatomical main group, therapeutic and chemical subgroup as well as BCS class of the fifteen considered drugs. (DOCX) [file pcbi.1005280.s005.docx]

#### S1 Table. Drug-specific annotations.

| **Drug** | **DILI-potential** | **Severity score** | **Anatomical main group** | **Therapeutic subgroup** | **Chemical subgroup** | **BCS class** |
| --- | --- | --- | --- | --- | --- | --- |
| APAP | Most-DILI-concern | 5 | Nervous system | Analgesics | Anilides | Class 4 |
| AD | Most-DILI-concern | 8 | Cardiovascular system | Cardiac therapy | Antiarrhythmics, class III | Class 2 |
| AZA | Less-DILI-concern | 3 | Antineoplastic and immunomodulating agents | Immunosuppressants | Other immunosuppressants | Class 4 |
| CPA | Less-DILI-concern | 5 | Antineoplastic and immunomodulating agents | Antineoplastic agents | Nitrogen mustard analogues | Class 3 |
| CSA | Less-DILI-concern | 2 | Antineoplastic and immunomodulating agents | Immunosuppressants | Calcineurin inhibitors | Class 2 |
| DFN | Most-DILI-concern | 7 | Musculo-skeletal system | Antiinflammatory and antirheumatic products | Acetic acid derivatives and related substances | Class 2 |
| ERY | Most-DILI-concern | 5 | Antiinfectives for systemic use | Antibacterials for systemic use | Macrolides | class 2 |
| FT | Most-DILI-concern | 8 | Antineoplastic and immunomodulating agents | Endocrine therapy | Anti-androgens | class 2 |
| HPL | Less-DILI-concern | 5 | Nervous system | Psycholeptics | Butyrophenone derivatives | class 2 |
| INH | Most-DILI-concern | 8 | Antiinfectives for systemic use | Antimycobacterials | Hydrazides | class 3 |
| PB | Less-DILI-concern | 3 | Nervous system | Antiepileptics | Barbiturates and derivatives | class 4 |
| PHE | Less-DILI-concern | 3 | Nervous system | Antiepileptics | Hydantoin derivatives | class 1 |
| RIF | Most-DILI-concern | 8 | Antiinfectives for systemic use | Antimycobacterials | Antibiotics | class 2 |
| SST | Less-DILI-concern | 3 | Cardiovascular system | Lipid modifying agents | HMG coa reductase inhibitors | class 2 |
| VPA | Most-DILI-concern | 8 | Nervous system | Antiepileptics | Fatty acid derivatives | class 3 |

DILI-potential, severity score, anatomical main group, therapeutic and chemical subgroup as well as BCS class of the fifteen considered drugs.
