# Supplementary material for: A Comparative Analysis of Drug-Induced Hepatotoxicity in Clinically Relevant Situations
Source: PLoS Comput Biol. 2017 Feb 2;13(2):e1005280. doi: 10.1371/journal.pcbi.1005280 (PMC5289425; doi:10.1371/journal.pcbi.1005280)
Supplement: S2 Table — Seventy-four toxicity lists representing key cellular processes were taken from QIAGENs Ingenuity Pathway Analysis (IPA, QIAGEN Redwood City, www.qiagen.com/ingenuity). (DOCX) [file pcbi.1005280.s006.docx]

#### S2 Table. Toxicity lists.

Seventy-four toxicity lists representing key cellular processes were taken from QIAGENs Ingenuity Pathway Analysis (IPA®, QIAGEN Redwood City, www.qiagen.com/ingenuity).

| **Toxicity list id** | **Toxicity list name** |
| --- | --- |
| TOX_LIST:01 | Anti-apoptosis |
| TOX_LIST:02 | Aryl hydrocarbon receptor signaling |
| TOX_LIST:03 | Biogenesis of mitochondria |
| TOX_LIST:04 | CAR/RXR activation |
| TOX_LIST:05 | Cardiac fibrosis |
| TOX_LIST:06 | Cardiac hypertrophy |
| TOX_LIST:07 | Cardiac necrosis/cell death |
| TOX_LIST:08 | Cell cycle G1/S checkpoint regulation |
| TOX_LIST:09 | Cell cycle G2/M DNA damage checkpoint regulation |
| TOX_LIST:10 | Cholesterol biosynthesis |
| TOX_LIST:11 | Cytochrome p450 - substrate is a eicosanoid |
| TOX_LIST:12 | Cytochrome p450 - substrate is a fatty acid |
| TOX_LIST:13 | Cytochrome p450 - substrate is a sterol |
| TOX_LIST:14 | Cytochrome p450 - substrate is a vitamin |
| TOX_LIST:15 | Cytochrome p450 - substrate is a xenobiotic |
| TOX_LIST:16 | Decreases depolarization of mitochondria and mitochondria membrane |
| TOX_LIST:17 | Decreases permeability transition of mitochondria and mitochondrial membrane |
| TOX_LIST:18 | Decreases respiration of mitochondria |
| TOX_LIST:19 | Decreases transmembrane potential of mitochondria and mitochondrial membrane |
| TOX_LIST:20 | Fatty acid metabolism |
| TOX_LIST:21 | FXR/RXR activation |
| TOX_LIST:22 | Genes associated with chronic allograft nephropathy |
| TOX_LIST:23 | Genes upregulated in response to proteinuria-induced oxidative stress in renal proximal tubule cells |
| TOX_LIST:24 | Glutathione depletion - CYP induction and reactive metabolites |
| TOX_LIST:25 | Glutathione depletion - hepatocellular hypertrophy |
| TOX_LIST:26 | Glutathione depletion - phase II reactions |
| TOX_LIST:27 | Hepatic cholestasis |
| TOX_LIST:28 | Hepatic fibrosis |
| TOX_LIST:29 | Hepatic stellate cell activation |
| TOX_LIST:30 | Hormone receptor regulated cholesterol metabolism |
| TOX_LIST:31 | Hypoxia-inducible factor signaling |
| TOX_LIST:32 | Increases bradycardia |
| TOX_LIST:33 | Increases cardiac dilation |
| TOX_LIST:34 | Increases cardiac dysfunction |
| TOX_LIST:35 | Increases cardiac proliferation |
| TOX_LIST:36 | Increases damage of mitochondria |
| TOX_LIST:37 | Increases depolarization of mitochondria and mitochondrial membrane |
| TOX_LIST:38 | Increases glomerular injury |
| TOX_LIST:39 | Increases heart failure |
| TOX_LIST:40 | Increases liver damage |
| TOX_LIST:41 | Increases liver hepatitis |
| TOX_LIST:42 | Increases liver hyperplasia/hyperproliferation |
| TOX_LIST:43 | Increases liver steatosis |
| TOX_LIST:44 | Increases permeability transition of mitochondria and mitochondrial membrane |
| TOX_LIST:45 | Increases renal damage |
| TOX_LIST:46 | Increases renal nephritis |
| TOX_LIST:47 | Increases renal proliferation |
| TOX_LIST:48 | Increases transmembrane potential of mitochondria and mitochondrial membrane |
| TOX_LIST:49 | Liver necrosis/cell death |
| TOX_LIST:50 | Liver proliferation |
| TOX_LIST:51 | LPS/IL-1 mediated inhibition of RXR function |
| TOX_LIST:52 | LXR/RXR activation |
| TOX_LIST:53 | Mechanism of gene regulation by peroxisome proliferations via PPARα |
| TOX_LIST:54 | Mitochondrial dysfunction |
| TOX_LIST:55 | Negative acute phase response proteins |
| TOX_LIST:56 | NF-κB signaling |
| TOX_LIST:57 | Nongenotoxic hepatocarcinogenicity biomarker panel |
| TOX_LIST:58 | NRF-2 mediated oxidative stress response |
| TOX_LIST:59 | Oxidative stress |
| TOX_LIST:60 | P53 signaling |
| TOX_LIST:61 | Positive acute phase response proteins |
| TOX_LIST:62 | PPARα / RXRα activation |
| TOX_LIST:63 | Primary glomerulonephritis biomarker panel |
| TOX_LIST:64 | Pro-apoptosis |
| TOX_LIST:65 | PXR/RXR activation |
| TOX_LIST:66 | RAR activation |
| TOX_LIST:67 | Renal glomerulus panel |
| TOX_LIST:68 | Renal necrosis/cell death |
| TOX_LIST:69 | Renal safety biomarker panel |
| TOX_LIST:70 | Swelling of mitochondria |
| TOX_LIST:71 | TGF-β signaling |
| TOX_LIST:72 | TR/RXR activation |
| TOX_LIST:73 | VDR/XRX activation |
| TOX_LIST:74 | Xenobiotic metabolism signaling |
