# Supplementary material for: A Comparative Analysis of Drug-Induced Hepatotoxicity in Clinically Relevant Situations
Source: PLoS Comput Biol. 2017 Feb 2;13(2):e1005280. doi: 10.1371/journal.pcbi.1005280 (PMC5289425; doi:10.1371/journal.pcbi.1005280)
Supplement: S3 Table — Administration route (intravenous (iv), or oral (po)), respective doses, number of subjects and health state. The experimental PK data were either used for establishment of the reference PBPK model (Reference) or for model validation (Validation). (DOCX) [file pcbi.1005280.s007.docx]

#### S3 Table. Experimental conditions.

Administration route (intravenous (iv), or oral (po)), respective doses, number of subjects and health state. The experimental PK data were either used for establishment of the reference PBPK model (Reference) or for model validation (Validation).

| **Drug** | **Route** | **Dose** | **Subjects** | **Health state** | **Model type** | **Reference** |
| --- | --- | --- | --- | --- | --- | --- |
| APAP | po | 1000 mg | 5 | Healthy | Reference | (1) |
| APAP | po | 20 mg/kg | 8 | Healthy | Validation | (2) |
| AD | iv | 400 mg | 7 | Sick | Reference | (3) |
| AD | po | 400 mg | 7 | Sick | Reference | (3) |
| AZA | iv | 100 mg | 15 | Sick | Validation | (4) |
| AZA | iv | 50 mg | 24 | Healthy | Reference | (5) |
| AZA | po | 100 mg | 10 | Healthy | Reference | (6) |
| CPA | iv | 800 mg | 12 | Sick | Reference | (7) |
| CPA | iv | 800 mg | 1 | Healthy | Validation | (8) |
| CPA | iv | 200 mg | 1 | Sick | Validation | (8) |
| CPA | po | 300 mg | 1 | Healthy | Reference | (9) |
| CSA | iv | 4 mg/kg | 1 | Sick | Reference | (10) |
| CSA | iv | 4 mg/kg | 1 | Healthy | Validation | (11) |
| CSA | po | 10 mg/kg | 1 | Sick | Reference | (10) |
| CSA | po | 10 mg/kg | 1 | Healthy | Validation | (11) |
| DFN | iv | 50 mg | 7 | Healthy | Reference | (12) |
| DFN | po | 50 mg | 7 | Healthy | Validation | (12) |
| DFN | po | 100 mg | 3 | Healthy | Reference | (13) |
| ERY | iv | 500 mg | 6 | Healthy | Reference | (14) |
| ERY-PED | po | 400 mg | 24 | Healthy | Reference | (15) |
| FT | po | 250 mg | 6 | Healthy | Reference | (16) |
| HPL | iv | 3.5 mg | 1 | Sick | Reference | (17) |
| HPL | po | 2 mg | 1 | Sick | Reference | (17) |
| INH | iv | 670 mg | 1 | Healthy | Reference | (18) |
| INH | iv | 681 mg | 1 | Healthy | Validation | (18) |
| INH | po | 300 mg | 8 | Healthy | Reference | (19) |
| INH | po | 300 mg | 8 | Healthy | Validation | (19) |
| PB | iv | 2.6 mg/kg | 6 | Healthy | Reference | (20) |
| PB | po | 2.9 mg/kg | 6 | Healthy | Reference | (20) |
| PB | po | 5.21 mg/kg | 1 | Healthy | Validation | (21) |
| PB | po | 4.33 mg/kg | 1 | Healthy | Validation | (21) |
| PHE | iv | 5 mg/kg | 2 | Healthy | Reference | (22) |
| PHE | po | 5 mg/kg | 2 | Healthy | Reference | (22) |
| PHE | po | 300 mg | 6 | Healthy | Validation | (23) |
| RIF | iv | 300 mg | 12 | Healthy | Reference | (24) |
| RIF | iv | 600 mg | 12 | Healthy | Validation | (24) |
| RIF | po | 150 mg | 8 | Healthy | Validation | (25) |
| RIF | po | 300 mg | 8 | Healthy | Validation | (25) |
| RIF | po | 450 mg | 8 | Healthy | Reference | (25) |
| RIF | po | 600 mg | 8 | Healthy | Validation | (25) |
| SST | po | 40 mg | 10 | Healthy | Reference | (26) |
| VPA | iv | 800 mg | 6 | Healthy | Reference | (27) |
| VPA | po | 800 mg | 6 | Healthy | Reference | (27) |
| VPA | po | 600 mg | 6 | Healthy | Validation | (28) |
| VPA | po | 1000 mg | 6 | Healthy | Validation | (29) |

**REFERENCES**

1. Shinoda S, Aoyama T, Aoyama Y, Tomioka S, Matsumoto Y, Ohe Y. Pharmacokinetics/pharmacodynamics of acetaminophen analgesia in Japanese patients with chronic pain. Biol Pharm Bull. 2007 Jan;30(1):157–61.

2. Prescott LF. Kinetics and metabolism of paracetamol and phenacetin. Br J Clin Pharmacol. 1980 Oct;10 Suppl 2(S2):291S–298S.

3. Andreasen F, Agerbaek H, Bjerregaard P, Gøtzsche H. Pharmacokinetics of amiodarone after intravenous and oral administration. Eur J Clin Pharmacol. 1981 Mar;19(4):293–9.

4. Odlind B, Hartvig P, Lindström B, Lönnerholm G, Tufveson G, Grefberg N. Serum azathioprine and 6-mercaptopurine levels and immunosuppressive activity after azathioprine in uremic patients. Int J Immunopharmacol. 1986 Jan;8(1):1–11.

5. Van Os EC, Zins BJ, Sandborn WJ, Mays DC, Tremaine WJ, Mahoney DW, et al. Azathioprine pharmacokinetics after intravenous, oral, delayed release oral and rectal foam administration. Gut. 1996 Jul 1;39(1):63–8.

6. Zins BJ, Sandborn WJ, McKinney J a, Mays DC, van Os EC, Tremaine WJ, et al. A dose-ranging study of azathioprine pharmacokinetics after single-dose administration of a delayed-release oral formulation. Vol. 37, Journal of clinical pharmacology. 1997.

7. Haubitz M, Bohnenstengel F, Brunkhorst R, Schwab M, Hofmann U, Busse D. Cyclophosphamide pharmacokinetics and dose requirements in patients with renal insufficiency. Kidney Int. 2002 Apr;61(4):1495–501.

8. Juma FD, Rogers HJ, Trounce JR. Effect of renal insufficiency on the pharmacokinetics of cyclophosphamide and some of its metabolites. Eur J Clin Pharmacol. 1981;19(6):443–51.

9. Juma FD, Rogers HJ, Trounce JR. Pharmacokinetics of cyclophosphamide and alkylating activity in man after intravenous and oral administration. Br J Clin Pharmacol. 1979 Sep;8(3):209–17.

10. Aweeka FT, Tomlanovich SJ, Prueksaritanont T, Gupta SK, Benet LZ. Pharmacokinetics of orally and intravenously administered cyclosporine in pre-kidney transplant patients. J Clin Pharmacol. 1994 Jan 8;34(1):60–7.

11. Gupta SK, Manfro RC, Tomlanovich SJ, Gambertoglio JG, Garovoy MR, Benet LZ. Effect of food on the pharmacokinetics of cyclosporine in healthy subjects following oral and intravenous administration. J Clin Pharmacol. 1990 Jul 8;30(7):643–53.

12. Willis JV, Kendall MJ, Flinn RM, Thornbill DP, Welling PG. The pharmacokinetics of diclofenc sodium following intravenous and oral administration. Eur J Clin Pharmacol. 1979;16(6):405–10.

13. Degen PH, Dieterle W, Schneider W, Theobald W, Sinterhauf U. Pharmacokinetics of Diclofenac and Five Metabolites After Single Doses in Healthy Volunteers and After Repeated Doses in Patients. Xenobiotica. 1988 Jan 30;18(12):1449–55.

14. Barre J, Mallat A, Rosenbaum J, Deforges L, Houin G, Dhumeaux D, et al. Pharmacokinetics of erythromycin in patients with severe cirrhosis. Respective influence of decreased serum binding and impaired liver metabolic capacity. Br J Clin Pharmacol. 1987 Jun;23(6):753–7.

15. Zakeri-Milani P, Ghanbarzadeh S, Lotfi poor F, Milani M, Valizadeh H. Pharmacokinetic Study of Two Macrolide Antibiotic Oral Suspensions Using an Optimized Bioassay Procedure. J Bioequiv Availab. 2010;2(5):111–5.

16. Anjum, Swan, Lambrecht, Radwanski, Cutler, Affrime, et al. Pharmacokinetics of flutamide in patients with renal insufficiency. Br J Clin Pharmacol. 2001 Dec 24;47(1):43–7.

17. Cheng YF, Paalzow LK, Bondesson U, Ekblom B, Eriksson K, Eriksson SO, et al. Pharmacokinetics of haloperidol in psychotic patients. Psychopharmacology (Berl). 1987;91(4):410–4.

18. Boxenbaum HG, Riegelman S. Determination of isoniazid and metabolites in biological fluids. J Pharm Sci. 1974 Aug;63(8):1191–7.

19. Bing C, Xiaomeia C, Jinhenga L. Gene dose effect of NAT2 variants on the pharmacokinetics of isoniazid and acetylisoniazid in healthy Chinese subjects. Drug Metabol Drug Interact. 2011 Jan 1;26(3):113–8.

20. Nelson E, Powell JR, Conrad K, Likes K, Byers J, Baker S, et al. Phenobarbital pharmacokinetics and bioavailability in adults. J Clin Pharmacol. 1982;22(2–3):141–8.

21. Boréus LO, Jalling B, Kållberg N. Phenobarbital metabolism in adults and in newborn infants. Acta Paediatr Scand. 1978 Mar;67(2):193–200.

22. Lund L, Alvan G, Berlin A, Alexanderson B. Pharmacokinetics of single and multiple doses of phenytoin in man. Eur J Clin Pharmacol. 1974;7(2):81–6.

23. Velpandian T, Jasuja R, Bhardwaj RK, Jaiswal J, Gupta SK. Piperine in food: interference in the pharmacokinetics of phenytoin. Eur J Drug Metab Pharmacokinet. 2001;26(4):241–7.

24. FDA. Food and Drug Administration. Drugs@FDA http://www.accessdata.fda.gov/drugsatfda_docs/label/2010/050420s073,050627s012lbl.pdf [Accessed 29 September 2015]. 2015;

25. Riess W, W R. The optimum dosage schedule for Rimactane. A Symp Rimactane. 1968;36–42.

26. Lilja JJ, Neuvonen M, Neuvonen PJ. Effects of regular consumption of grapefruit juice on the pharmacokinetics of simvastatin. Br J Clin Pharmacol. 2004 Jul;58(1):56–60.

27. Perucca E, Gatti G, Frigo GM, Crema A, Calzetti S, Visintini D. Disposition of sodium valproate in epileptic patients. Br J Clin Pharmacol. 1978 Jun 14;5(6):495–9.

28. Gugler R, Schell A, Eichelbaum M, Fröscher W, Schulz HU. Disposition of valproic acid in man. Eur J Clin Pharmacol. 1977 Oct 14;12(2):125–32.

29. Bialer M, Hussein Z, Raz I, Abramsky O, Herishanu Y, Pachys F. Pharmacokinetics of valproic acid in volunteers after a single dose study. Biopharm Drug Dispos. 1985;6(1):33–42.
