# Supplementary material for: A Comparative Analysis of Drug-Induced Hepatotoxicity in Clinically Relevant Situations
Source: PLoS Comput Biol. 2017 Feb 2;13(2):e1005280. doi: 10.1371/journal.pcbi.1005280 (PMC5289425; doi:10.1371/journal.pcbi.1005280)
Supplement: S4 Table — Metabolic and active drug transport processes either consist of the metabolic enzyme and the corresponding metabolite or of the transporter and the corresponding transporter type (efflux, influx). Kinetic parameters Km and vmax were used to characterize the kinetic behavior of active processes. A liver plasma clearance of 11.5 ml/min/kg was estimated for the clearance of 2-hydroxy-FT. For INH, NAT2 polymorphism was considered by estimating two different vmax values to best describe clinical data available for fast and slow metabolizer [66,67]. (DOCX) [file pcbi.1005280.s008.docx]

#### S4 Table. Active drug transport and metabolic processes.

Metabolic and active drug transport processes either consist of the metabolic enzyme and the corresponding metabolite or of the transporter and the corresponding transporter type (efflux, influx). Kinetic parameters Km and vmax were used to characterize the kinetic behavior of active processes. A liver plasma clearance of 11.5 ml/min/kg was estimated for the clearance of 2-hydroxy-FT. For INH, NAT2 polymorphism was considered by estimating two different vmax values to best describe clinical data available for fast and slow metabolizer (1,2).

| **ID** | **Drug / Metabolite** | **Metabolite /**  **Transporter type** | **Metabolic enzyme /**  **Transporter** | **Km**  **[µmol/l]** | **vmax**  **[µmol/l/min]** | **Reference** |
| --- | --- | --- | --- | --- | --- | --- |
| 1 | APAP | APAP-glucuronide | UGT1A9 | 19742* | 5343.10* | (3) |
| 1 | APAP | APAP-sulfate | SULT1A1 | 9963.80* | 192.97* | (4) |
| 1 | APAP | NAPQI | CYP2E1 | 41.26* | 2.40* | (5) |
| 1 | APAP-glucuronide | Efflux | ABCG2 | 96.30* | 21.80* | (6) |
| 1 | APAP-sulfate | Efflux | ABCG2 | 94.49* | 1984.41* | (6) |
| 1 | NAPQI | APAP cystein | GSTT1 | 25.00* | 569.00* | (5) |
| 2 | AD | Desethyl-AD | CYP1A1 | 15.90 | 30.00* | (7) |
| 3 | 6-MP | 6-thiouric acid | XO | 41.50* | 410.00* | (8) |
| 3 | AZA | 6-MP | GSTA1 | 7.00* | 60.00* | (9) |
| 4 | CPA | 4-hydroxy-CPA | CYP2B6 | 91.00* | 3.70* | (10–12) |
| 5 | CSA | M1, M9, M4n | CYP3A4 | 2.30 | 108.00* | (13,14) |
| 6 | DFN | 4-hydroxy-DFN | CYP2C9 | 9.00 | 530.00* | (15,16) |
| 7 | ERY | N-desmethyl ERY | CYP3A4 | 46.00 | 23.00* | (17,18) |
| 7 | ERY-PED | N-desmethyl ERY | CYP3A4 | 46.00 | 23.00* | (17,18) |
| 8 | FT | 2-hydroxy-FT | CYP1A2 | 10.00* | 17.30* | (19) |
| 9 | HPL | Reduced HPL,  HPL pyridinium derivative,  4-Fluorobenzoylpropionic acid | CYP3A4 | 34.00 | 47.00* | (20,21) |
| 10 | Acetyl-INH | Isonicotinic acid,  Acetylhydrazine | NAAA | 500.00* | 30.00* | (22) |
| 10 | INH | Acetyl-INH | NAT2 | 1950.00* | 400.00*^,++^ | (22) |
| 10 | INH | Acetyl-INH | NAT2 | 1950.00* | 70.00*^,+^ | (22) |
| 10 | INH | Isonicotinic acid,  Hydrazine | NAAA | 2000.00* | 4.00* | (22) |
| 11 | PB | P-hydroxy-PB | CYP2C19 | 147.00* | 0.27* | (14) |
| 12 | PHE | 5-(p-hydroxyphenyl-),  5-phenylhydantoin | CYP2C19 | 16.90 | 2.40* | (23,24) |
| 13 | RIF | 25-desacetyl-RIF | CES2 | 39.20* | 1.95* | (25–27) |
| 14 | SST | SST-acid | CES2 | 10.00 | 223.92* | (28–30) |
| 14 | SST | 6-hydroxy-SST,  3-hydroxy-SST,  6-exomethylene | CYP3A4 | 10.00 | 10.25* | (28–30) |
| 14 | SST-acid | SST-acid-metabolites | CYP3A4 | 10.00 | 223.90* | (28–30) |
| 14 | SST-acid | Influx | OATP1B1 | 10.00 | 276.00* | (29,31) |
| 15 | VPA | Hydroxy-VPA | CYP2C9 | 40.00* | 0.90* | (14,32) |
| 15 | VPA | VPA-β-glucuronide | UGT1A8 | 60.00* | 0.90* | (14,33) |

* Estimated ^++^ Fast metabolizer ^+^ Slow metabolizer

**REFERENCES**

1. Boxenbaum HG, Riegelman S. Determination of isoniazid and metabolites in biological fluids. J Pharm Sci. 1974 Aug;63(8):1191–7.

2. Bing C, Xiaomeia C, Jinhenga L. Gene dose effect of NAT2 variants on the pharmacokinetics of isoniazid and acetylisoniazid in healthy Chinese subjects. Drug Metabol Drug Interact. 2011 Jan 1;26(3):113–8.

3. Mutlib AE, Goosen TC, Bauman JN, Williams JA, Kulkarni S, Kostrubsky S. Kinetics of acetaminophen glucuronidation by UDP-glucuronosyltransferases 1A1, 1A6, 1A9 and 2B15. Potential implications in acetaminophen-induced hepatotoxicity. Chem Res Toxicol. 2006;19(5):701–9.

4. Riches Z, Bloomer JC, Coughtrie MWH. Comparison of 2-aminophenol and 4-nitrophenol as in vitro probe substrates for the major human hepatic sulfotransferase, SULT1A1, demonstrates improved selectivity with 2-aminophenol. Biochem Pharmacol. 2007;74(2):352–8.

5. Shinoda S, Aoyama T, Aoyama Y, Tomioka S, Matsumoto Y, Ohe Y. Pharmacokinetics/pharmacodynamics of acetaminophen analgesia in Japanese patients with chronic pain. Biol Pharm Bull. 2007 Jan;30(1):157–61.

6. Mazaleuskaya LL, Sangkuhl K, Thorn CF, FitzGerald GA, Altman RB, Klein TE. PharmGKB summary: pathways of acetaminophen metabolism at the therapeutic versus toxic doses. Pharmacogenet Genomics. 2015 Aug;25(8):416–26.

7. Elsherbiny ME, El-Kadi AOS, Brocks DR. The metabolism of amiodarone by various CYP isoenzymes of human and rat, and the inhibitory influence of ketoconazole. J Pharm Pharm Sci. 2008 Jan;11(1):147–59.

8. Aberra FN, Lichtenstein GR. Review article: monitoring of immunomodulators in inflammatory bowel disease. Aliment Pharmacol Ther. 2005 Feb 15;21(4):307–19.

9. Kaplowitz N, Kuhlenkamp J. Inhibition of hepatic metabolism of azathioprine in vivo. Gastroenterology. 1978 Jan;74(1):90–2.

10. Huang Z, Roy P, Waxman DJ. Role of human liver microsomal CYP3A4 and CYP2B6 in catalyzing N-dechloroethylation of cyclophosphamide and ifosfamide. Biochem Pharmacol. 2000;59(99):961–72.

11. Gervot L, Rochat B, Gautier JC, Bohnenstengel F, Kroemer H, de Berardinis V, et al. Human CYP2B6: expression, inducibility and catalytic activities. Pharmacogenetics. 1999 Jun;9(3):295–306.

12. McDonald GB, Slattery JT, Bouvier ME, Ren S, Batchelder AL, Kalhorn TF, et al. Cyclophosphamide metabolism, liver toxicity, and mortality following hematopoietic stem cell transplantation. Blood. 2003;101(5):2043–8.

13. Vickers AE, Meyer E, Dannecker R, Keller B, Tynes RE, Maurer G. Human liver cytochrome P4503A biotransformation of the cyclosporin derivative SDZ IMM 125. Drug Metab Dispos. 1995 Mar;23(3):321–6.

14. Wishart DS, Knox C, Guo AC, Shrivastava S, Hassanali M, Stothard P, et al. DrugBank: a comprehensive resource for in silico drug discovery and exploration. Nucleic Acids Res. 2006 Jan 1;34(Database issue):D668–72.

15. Leemann T, Transon C, Dayer P. Cytochrome P450TB (CYP2C): a major monooxygenase catalyzing diclofenac 4’-hydroxylation in human liver. Life Sci. 1993;52(July):29–34.

16. Bort R, Macé K, Boobis A, Gómez-Lechón MJ, Pfeifer A, Castell J. Hepatic metabolism of diclofenac: role of human CYP in the minor oxidative pathways. Biochem Pharmacol. 1999 Sep 1;58(5):787–96.

17. Wang RW, Newton DJ, Scheri TD, Lu AY. Human cytochrome P450 3A4-catalyzed testosterone 6 beta-hydroxylation and erythromycin N-demethylation. Competition during catalysis. Drug Metab Dispos. 1997;25(4):502–7.

18. Zhang X, Jones DR, Hall SD. Prediction of the effect of erythromycin, diltiazem, and their metabolites, alone and in combination, on CYP3A4 inhibition. Drug Metab Dispos. 2009;37(1):150–60.

19. Shet MS, McPhaul M, Fisher CW, Stallings NR, Estabrook RW. Metabolism of the antiandrogenic drug (Flutamide) by human CYP1A2. Drug Metab Dispos. 1997 Nov;25(11):1298–303.

20. Avent KM, DeVoss JJ, Gillam EMJ. Cytochrome P450-mediated metabolism of haloperidol and reduced haloperidol to pyridinium metabolites. Chem Res Toxicol. 2006;19(7):914–20.

21. Froemming JS, Lam YW, Jann MW, Davis CM. Pharmacokinetics of haloperidol. Clin Pharmacokinet. 1989;17(6):396–423.

22. Ellard GA, Gammon PT. Pharmacokinetics of isoniazid metabolism in man. J Pharmacokinet Biopharm. 1976 Apr;4(2):83–113.

23. Cuttle L, Munns a. J, Hogg N a., Scott JR, Hooper WD, Dickinson RG, et al. Phenytoin metabolism by human cytochrome P450: Involvement of P450 3A and 2C forms in secondary metabolism and drug-protein adduct formation. Drug Metab Dispos. 2000;28(8):945–50.

24. Yukawa E, Mamiya K. Effect of CYP2C19 genetic polymorphism on pharmacokinetics of phenytoin and phenobarbital in Japanese epileptic patients using Non-linear Mixed Effects Model approach. J Clin Pharm Ther. 2006 Jun;31(3):275–82.

25. Sousa M, Pozniak A, Boffito M. Pharmacokinetics and pharmacodynamics of drug interactions involving rifampicin, rifabutin and antimalarial drugs. J Antimicrob Chemother. 2008 Nov;62(5):872–8.

26. Song SH, Chang HE, Jun SH, Park KU, Lee JH, Lee E-M, et al. Relationship between CES2 genetic variations and rifampicin metabolism. J Antimicrob Chemother. 2013 Jun;68(6):1281–4.

27. Jamis-Dow CA, Katki AG, Collins JM, Klecker RW. Rifampin and rifabutin and their metabolism by human liver esterases. Xenobiotica. 1997 Oct 22;27(10):1015–24.

28. Lilja JJ, Neuvonen M, Neuvonen PJ. Effects of regular consumption of grapefruit juice on the pharmacokinetics of simvastatin. Br J Clin Pharmacol. 2004 Jul;58(1):56–60.

29. Lippert J, Brosch M, von Kampen O, Meyer M, Siegmund H-U, Schafmayer C, et al. A Mechanistic, Model-Based Approach to Safety Assessment in Clinical Development. CPT Pharmacometrics Syst Pharmacol. 2013 Nov;1(11):e13.

30. García MJ, Reinoso RF, Sánchez Navarro A, Prous JR. Clinical pharmacokinetics of statins. Methods Find Exp Clin Pharmacol. 2003;25(6):457–81.

31. Kameyama Y, Yamashita K, Kobayashi K, Hosokawa M, Chiba K. Functional characterization of SLCO1B1 (OATP-C) variants, SLCO1B1*5, SLCO1B1*15 and SLCO1B1*15+C1007G, by using transient expression systems of HeLa and HEK293 cells. Pharmacogenet Genomics. 2005 Jul;15(7):513–22.

32. Kiang TKL, Ho PC, Anari MR, Tong V, Abbott FS, Chang TKH. Contribution of CYP2C9, CYP2A6, and CYP2B6 to Valproic Acid Metabolism in Hepatic Microsomes from Individuals with the CYP2C9*1/*1 Genotype. Toxicol Sci. 2006 Dec 31;94(2):261–71.

33. Ethell BT, Anderson GD, Burchell B. The effect of valproic acid on drug and steroid glucuronidation by expressed human UDP-glucuronosyltransferases. Biochem Pharmacol. 2003 May 1;65(9):1441–9.
