# Supplementary material for: A Comparative Analysis of Drug-Induced Hepatotoxicity in Clinically Relevant Situations
Source: PLoS Comput Biol. 2017 Feb 2;13(2):e1005280. doi: 10.1371/journal.pcbi.1005280 (PMC5289425; doi:10.1371/journal.pcbi.1005280)
Supplement: S5 Table — Renal and biliary clearance processes of the developed PBPK models. (DOCX) [file pcbi.1005280.s009.docx]

#### S5 Table. Renal and biliary clearance processes.

Renal and biliary clearance processes of the developed PBPK models.

| **ID** | **Drug / Metabolite** | **Route** | **Process type** | **Clearance** | **Reference** |
| --- | --- | --- | --- | --- | --- |
| 1 | APAP | Renal | Kidney plasma clearance | 13.62 ml/h/kg | (1) |
| 1 | APAP-cysteine | Renal | Kidney plasma clearance | 300.00 ml/h/kg | (1) |
| 1 | APAP-glucuronide | Renal | Kidney plasma clearance | 126.00 ml/h/kg | (1) |
| 1 | APAP-sulfate | Renal | Kidney plasma clearance | 10.12 ml/h/kg | (1) |
| 1 | NAPQI | Renal | Kidney plasma clearance | 120.00 ml/h/kg | (2) |
| 4 | CPA | Renal | Kidney plasma clearance | 15.30 ml/h/kg | (3) |
| 4 | CPA | Biliary | Biliary plasma clearance | 3.90 ml/h/kg | (3) |
| 6 | DFN | Renal | Kidney plasma clearance | 16.80 ml/h/kg | (3) |
| 7 | ERY | Renal | Kidney plasma clearance | 61.00 ml/h/kg | (4) |
| 7 | ERY-PED | Renal | Kidney plasma clearance | 61.00 ml/h/kg | (5) |
| 8 | 2-hydroxy-FT | Renal | Kidney plasma clearance | 0.26 ml/h/kg | (6) |
| 8 | FT | Renal | Kidney plasma clearance | 5.22 ml/h/kg | (6) |
| 9 | HPL | Renal | Kidney plasma clearance | 4.80 ml/h/kg | (7) |
| 10 | INH | Renal | Tubular secretion – MM kinetics  Michaelis Menten kinetics | Km = 300.00 µmol/l  vmax = 45.69 µmol/l/min | (8) |
| 10 | Acetyl-INH | Renal | Tubular secretion – MM kinetics  Michaelis Menten kinetics | Km = 20.00 µmol/l  vmax = 0.69 µmol/l/min | (8) |
| 11 | PB | Renal | Kidney plasma clearance | 0.99 ml/h/kg | (9) |
| 12 | PHE | Renal | Tubular secretion – First order | 0.22 l/h | (10) |
| 13 | RIF | Renal | Kidney plasma clearance | 16.80 ml/h/kg | (11) |
| 14 | SST | Renal | Kidney plasma clearance | 420.00 ml/h/kg | (12) |
| 15 | VPA | Renal | Kidney plasma clearance | 0.30 ml/h/kg | (13) |

**REFERENCES**

1. Critchley J a, Nimmo GR, Gregson C a, Woolhouse NM, Prescott LF. Inter-subject and ethnic differences in paracetamol metabolism. Br J Clin Pharmacol. 1986;22(6):649–57.

2. Krauss M, Schaller S, Borchers S, Findeisen R, Lippert J, Kuepfer L. Integrating Cellular Metabolism into a Multiscale Whole-Body Model. Papin JA, editor. PLoS Comput Biol. 2012 Oct 25;8(10):e1002750.

3. Wishart DS, Knox C, Guo AC, Shrivastava S, Hassanali M, Stothard P, et al. DrugBank: a comprehensive resource for in silico drug discovery and exploration. Nucleic Acids Res. 2006 Jan 1;34(Database issue):D668–72.

4. FDA. Food and Drug Administration. Drugs@FDA http://www.accessdata.fda.gov/drugsatfda_docs/label/2008/050609s025lbl.pdf [Accessed 26 August 2015]. 2015;

5. Periti P, Mazzei T, Mini E, Novelli A. Clinical pharmacokinetic properties of the macrolide antibiotics. Effects of age and various pathophysiological states (Part I). Clin Pharmacokinet. 1989 Apr;16(4):193–214.

6. Anjum, Swan, Lambrecht, Radwanski, Cutler, Affrime, et al. Pharmacokinetics of flutamide in patients with renal insufficiency. Br J Clin Pharmacol. 2001 Dec 24;47(1):43–7.

7. Froemming JS, Lam YW, Jann MW, Davis CM. Pharmacokinetics of haloperidol. Clin Pharmacokinet. 1989;17(6):396–423.

8. Mitchell JR, Thorgeirsson UP, Black M, Timbrell JA, Snodgrass WR, Potter WZ, et al. Increased incidence of isoniazid hepatitis in rapid acetylators: possible relation to hydranize metabolites. Clin Pharmacol Ther. 1975 Jul;18(1):70–9.

9. Boréus LO, Jalling B, Kållberg N. Phenobarbital metabolism in adults and in newborn infants. Acta Paediatr Scand. 1978 Mar;67(2):193–200.

10. Borgå O, Hoppel C, Odar-Cederlöf I, Garle M. Plasma levels and renal excretion of phenytoin and its metabolites in patients with renal failure. Clin Pharmacol Ther. 1979 Sep;26(3):306–14.

11. FDA. Food and Drug Administration. Drugs@FDA http://www.accessdata.fda.gov/drugsatfda_docs/label/2010/050420s073,050627s012lbl.pdf [Accessed 29 September 2015]. 2015;

12. García MJ, Reinoso RF, Sánchez Navarro A, Prous JR. Clinical pharmacokinetics of statins. Methods Find Exp Clin Pharmacol. 2003;25(6):457–81.

13. Gugler R, Schell A, Eichelbaum M, Fröscher W, Schulz HU. Disposition of valproic acid in man. Eur J Clin Pharmacol. 1977 Oct 14;12(2):125–32.
