# Supplementary material for: A Comparative Analysis of Drug-Induced Hepatotoxicity in Clinically Relevant Situations
Source: PLoS Comput Biol. 2017 Feb 2;13(2):e1005280. doi: 10.1371/journal.pcbi.1005280 (PMC5289425; doi:10.1371/journal.pcbi.1005280)
Supplement: S6 Table — Intestinal permeability values for all drugs and their metabolites. Some intestinal permeability values originally provided by the modeling software [60] (Initial intestinal permeability) were slightly adjusted (Intestinal permeability used in model) to best describe the experimental data for oral administration. (DOCX) [file pcbi.1005280.s010.docx]

#### S6 Table. Intestinal permeabilities.

Intestinal permeability values for all drugs and their metabolites. Some intestinal permeability values originally provided by the modeling software (Initial intestinal permeability) were slightly adjusted (Intestinal permeability used in model) to best describe the experimental data for oral administration.

| **ID** | **Drug / Metabolite** | **Initial intestinal permeability**  **[cm/min]** | **Intestinal permeability used in model**  **[cm/min]** |
| --- | --- | --- | --- |
| 1 | APAP | 5.33E-06 | 2.95E-05 |
| 1 | APAP-cysteine | 6.01E-07 | 6.01E-07 |
| 1 | APAP-glucuronidid | 8.06E-09 | 8.06E-09 |
| 1 | APAP-sulfate | 1.11E-07 | 1.11E-07 |
| 1 | NAPQI | 3.32E-06 | 3.32E-06 |
| 2 | AD | 8.64E-04 | 2.30E-04 |
| 3 | 6-MP | 1.71E-04 | 1.71E-04 |
| 3 | AZA | 2.04E-07 | 9.04E-04 |
| 4 | CPA | 3.08E-06 | 5.08E-05 |
| 5 | CSA | 1.67E-06 | 4.80E-04 |
| 6 | DFN | 3.13E-03 | 6.00E-03 |
| 7 | ERY | 2.33E-06 | 2.33E-06 |
| 7 | ERY-PED | 6.81E-06 | 6.50E-04 |
| 8 | 2-hydroxy FT | 3.65E-05 | 3.65E-05 |
| 8 | FT | 4.64E-04 | 1.85E-04 |
| 9 | HPL | 2.69E-04 | 4.69E-04 |
| 10 | Acetyl-INH | 1.46E-07 | 1.46E-07 |
| 10 | INH | 8.24E-07 | 2.00E-05 |
| 11 | PB | 4.81E-05 | 7.00E-05 |
| 12 | PHE | 4.51E-05 | 9.00E-05 |
| 13 | RIF | 1.03E-06 | 1.12E-04 |
| 14 | SST | 1.22E-03 | 5.90E-04 |
| 14 | SST-acid | 4.20E-04 | 6.90E-05 |
| 15 | Hydroxyl-VPA | 5.03E-05 | 5.03E-05 |
| 15 | VPA | 6.21E-04 | 6.21E-04 |
| 15 | VPA-β-glucuronide | 6.00E-07 | 6.00E-07 |
