# Supplementary material for: A Comparative Analysis of Drug-Induced Hepatotoxicity in Clinically Relevant Situations
Source: PLoS Comput Biol. 2017 Feb 2;13(2):e1005280. doi: 10.1371/journal.pcbi.1005280 (PMC5289425; doi:10.1371/journal.pcbi.1005280)
Supplement: S7 Table — Different calculation methods used in the established PBPK models to calculate intracellular to plasma partition coefficients as well as permeability values between interstitial and cellular space. The calculation methods are provided in the modeling software [60]. (DOCX) [file pcbi.1005280.s011.docx]

#### S7 Table. Calculation methods for partition coefficients and cellular permeabilities.

Different calculation methods used in the established PBPK models to calculate intracellular to plasma partition coefficients as well as permeabilities between interstitial and cellular space. The calculation methods are provided in the modeling software.

| **ID** | **Drug / Metabolite** | **Partition coefficients** | **Cellular permeabilities** |
| --- | --- | --- | --- |
| 1 | APAP | Schmitt | Charge dependent Schmitt |
| 1 | APAP-cysteine | Schmitt | Charge dependent Schmitt |
| 1 | APAP-glucuronidid | Schmitt | Charge dependent Schmitt |
| 1 | APAP-sulfate | Schmitt | Charge dependent Schmitt |
| 1 | NAPQI | Schmitt | Charge dependent Schmitt |
| 2 | AD | Schmitt | PK-Sim Standard |
| 3 | 6-MP | Schmitt | PK-Sim Standard |
| 3 | AZA | PK-Sim Standard | PK-Sim Standard |
| 4 | CPA | Schmitt | PK-Sim Standard |
| 5 | CSA | PK-Sim Standard | PK-Sim Standard |
| 6 | DFN | Schmitt | PK-Sim Standard |
| 7 | ERY | PK-Sim Standard | PK-Sim Standard |
| 7 | ERY-PED | PK-Sim Standard | PK-Sim Standard |
| 8 | 2-hydroxy-FT | Rodgers and Rowland | PK-Sim Standard |
| 8 | FT | Rodgers and Rowland | PK-Sim Standard |
| 9 | HPL | Schmitt | PK-Sim Standard |
| 10 | Acetyl-INH | Schmitt | Charge dependent Schmitt |
| 10 | INH | Schmitt | Charge dependent Schmitt |
| 11 | PB | PK-Sim Standard | PK-Sim Standard |
| 12 | PHE | Rodgers and Rowland | PK-Sim Standard |
| 13 | RIF | Schmitt | PK-Sim Standard |
| 14 | SST | PK-Sim Standard | PK-Sim Standard |
| 14 | SST-acid | PK-Sim Standard | PK-Sim Standard |
| 15 | Hydroxyl-VPA | Schmitt | PK-Sim Standard |
| 15 | VPA | Schmitt | PK-Sim Standard |
| 15 | VPA-β-glucuronide | Schmitt | PK-Sim Standard |
