# Supplementary material for: A Comparative Analysis of Drug-Induced Hepatotoxicity in Clinically Relevant Situations
Source: PLoS Comput Biol. 2017 Feb 2;13(2):e1005280. doi: 10.1371/journal.pcbi.1005280 (PMC5289425; doi:10.1371/journal.pcbi.1005280)
Supplement: S8 Table — Toxic dose levels for the fifteen drugs were identified by database and literature screening. To determine a toxic dose for SST and FT, toxic rat doses [21] were scaled since no appropriate doses were found in literature. (DOCX) [file pcbi.1005280.s012.docx]

#### S8 Table. Toxic dose levels.

Toxic dose levels for the fifteen drugs were identified by database and literature screening. To determine a toxic dose for SST and FT, toxic rat doses (1) were scaled since no appropriate doses were found in literature.

| **Drug** | **Mean toxic dose**  **[mg/kg]** | **References** |
| --- | --- | --- |
| APAP | 465.00 | (2,3) |
| AD | 58.70 | (2,3) |
| AZA | 46.40 | (4) |
| CPA | 221.90 | (5) |
| CSA | 134.70 | (2) |
| DFN | 13.70 | (6) |
| ERY | 39.00 | (3,7) |
| FT | 21.20 | Scaled |
| HPL | 378.20 | (2,8) |
| INH | 203.30 | (2) |
| PB | 68.50 | (2) |
| PHE | 153.00 | (2) |
| RIF | 99.00 | (2,9) |
| SST | 56.60 | Scaled |
| VPA | 606.00 | (2) |

**REFERENCES**

1. Igarashi Y, Nakatsu N, Yamashita T, Ono A, Ohno Y, Urushidani T, et al. Open TG-GATEs: a large-scale toxicogenomics database. Nucleic Acids Res. 2015 Jan 13;43(Database issue):D921-7.

2. Clemedson C, Kolman A, Forsby A. The integrated acute systemic toxicity project (ACuteTox) for the optimisation and validation of alternative in vitro tests. Altern Lab Anim. 2007 Mar;35(1):33–8.

3. Hoofnagle JH, Serrano J, Knoben JE, Navarro VJ. LiverTox: a website on drug-induced liver injury. Hepatology. 2013 Mar;57(3):873–4.

4. Gregoriano C, Ceschi A, Rauber-Lüthy C, Kupferschmidt H, Banner NR, Krähenbühl S, et al. Acute thiopurine overdose: Analysis of reports to a national poison centre 1995-2013. PLoS One. 2014 Jan;9(1):e86390.

5. Aguiar Bujanda D, Cabrera Suárez MÁA, Bohn Sarmiento U, Aguiar Morales J. Successful recovery after accidental overdose of cyclophosphamide. Ann Oncol. 2006 Aug;17(8):1334.

6. von Mach M a, Hermanns-Clausen M, Koch I, Hengstler JG, Lauterbach M, Kaes J, et al. Experiences of a poison center network with renal insufficiency in acetaminophen overdose: an analysis of 17 cases. Clin Toxicol (Phila). 2005;43(1):31–7.

7. Tenenbein MSM, Tenenbein MSM. Acute pancreatitis due to erythromycin overdose. Pediatr Emerg Care. 2005 Oct;21(10):675–6.

8. Henderson RA, Lane S, Henry JA. Life-threatening ventricular arrhythmia (torsades de pointes) after haloperidol overdose. Hum Exp Toxicol. 1991 Jan;10(1):59–62.

9. Spalding CT, Buss WC. Toxic overdose of isoniazid, rifampicin and ethambutol. Eur J Clin Pharmacol. 1986 Jan;30(3):381–2.
