# Supplementary material for: A Comparative Analysis of Drug-Induced Hepatotoxicity in Clinically Relevant Situations
Source: PLoS Comput Biol. 2017 Feb 2;13(2):e1005280. doi: 10.1371/journal.pcbi.1005280 (PMC5289425; doi:10.1371/journal.pcbi.1005280)
Supplement: S9 Table — Bioavailability values after 24 h calculated by use of the modeling software PK-Sim [60] (DOCX) [file pcbi.1005280.s013.docx]

#### S9 Table. Bioavailability values.

Bioavailability values after 24 h calculated by use of the modeling software PK-Sim®

| **Drug** | **Bioavailability [%]** |
| --- | --- |
| APAP | 92.0 |
| AD | 59.0 |
| AZA | 18.0 |
| CPA | 98.0 |
| CSA | 18.0 |
| DFN | 79.0 |
| ERY | 80.0 |
| FT | 84.0 |
| HPL | 93.0 |
| INH | 94.0 |
| PB | 98.0 |
| PHE | 76.0 |
| RIF | 94.0 |
| SST | 44.0 |
| VPA | 99.0 |
