# Supplementary material for: A Comparative Analysis of Drug-Induced Hepatotoxicity in Clinically Relevant Situations
Source: PLoS Comput Biol. 2017 Feb 2;13(2):e1005280. doi: 10.1371/journal.pcbi.1005280 (PMC5289425; doi:10.1371/journal.pcbi.1005280)
Supplement: S1 Text — (DOCX) [file pcbi.1005280.s018.docx]

# SUPPLEMENTARY MATERIALS & METHODS

## Software

Transcriptome analysis was performed in the statistical language R (version 3.1.0, 2014, R Core Team, <http://www.R-project.org>). All PBPK models were built by use of the software PK-Sim® (1–5) (version 6.0, Bayer Technology Services, GmbH, Leverkusen, Germany) and MoBi® (version 3.4, Bayer Technology Services). PK-Sim® as well as MoBi® are freely available for academic use. Results of the principal component analysis and the comparative toxicity analysis were visualized by use of the web tool ClustVis (6).

## Key cellular processes

Seventy-four hand-curated toxicity lists were extracted from QIAGENs Ingenuity Pathway Analysis (IPA®, QIAGEN Redwood City, www.qiagen.com/ingenuity) (S2 Table) to represent key cellular processes. These toxicity lists consist of gene sets contributing to a specific type of toxicity and were generated based on crucial biological processes and key toxicological responses. Furthermore, all genes associated to a certain toxicity list were functionally classified into one of the following groups: Cytokine, growth factor, metabolic enzyme, G-protein coupled receptor, ion channel, kinase, ligand-dependent nuclear receptor, other, peptidase, phosphatase, transcription regulator, translation regulator, transmembrane protein or transporter.

## Therapeutic and toxic dose levels

The therapeutic doses were taken from the clinical studies used to develop the PBPK models for oral administration (S3 Table). The databases from LiverTox® (7) and ACuteTox (8) as well as literature (9–13) were screened to set a toxic dose level for the fifteen hepatotoxic drugs (S8 Table). During the screening process, only sub-lethal doses were used while lethal doses were neglected. In the case of multiple identified doses, the mean value was set as therapeutic and toxic dose, respectively. Toxic doses for SST and FT were scaled from minimum toxic doses observed in rats (14) since no appropriate doses could be found in literature. Thereby, a mean scaling factor was computed between minimum toxic doses from rats (14) and mean toxic doses found in humans for all remaining drugs.

## Set of drugs

In this study, fifteen hepatotoxic drugs (APAP, AD, AZA, CPA, CSA, DFN, ERY, FT, HPL, INH, PB, PHE, RIF, and SST) were analyzed. The drugs have been selected based on pharmaceutical and chemical diversity, physicochemical properties, availability of in vitro toxicity data and experimental drug concentration-time profiles as well as concern for DILI (S1 Table). The drugs were categorized into drugs with most or less DILI concern (15,16). Assigned severity scores were between two and eight (15,16). The World Health Organization’s Anatomical Therapeutic Chemical (ATC) classification system (17) was used to separate the drugs into different groups according to the organ or system on which they act and their pharmacological and chemical properties (ATC index available at <http://www.whocc.no/atc_ddd_index/>, [Accessed 2015 November 27]) (S1 Table). The Biopharmaceutics Classification System (BCS) (18) was used to classify the drugs based on their solubility and permeability properties (S1 Table). The BCS classification of drugs was obtained from the Therapeutic System Research Laboratories website (http://www.tsrlinc.net/search.cfm, [Accessed 2015 October 30]) and from literature (19,20).

## In vitro toxicity data

Time-series gene expression profiles from Open TG-GATEs (14) (ArrayExpress accession numbers: E-MTAB-797, E-MTAB-798, E-MTAB-799), a large-scale toxicogenomics database, were used to obtain quantitative drug response data measured in human and rat hepatocytes as well as in rat livers. Gene expression was measured for three exposed concentrations (low, middle, high) after three exposure durations (2 h, 8 h and 24 h) in the in vitro study and after four exposure durations (3 h, 6 h, 9 h, 24 h) in the in vivo study, respectively, by use of Affymetrix Human Genome U133 Plus 2.0 and Affymetrix Rat Genome 230 2.0 GeneChip arrays. Data normalization was performed by applying the GC-RMA method (21). Probe sets on the chip were mapped to Entrez Gene IDs using BrainArray custom CDF files (version 19.0.0, ENTREZG) (22). Fold change values were calculated to indicate gene expression changes compared to the time-matched control (14). For each in vitro measurement in primary human hepatocytes, differential gene expression analysis was performed (absolute fold change > 1.5, corrected p-value < 0.01) by linear models using limma (23) and hypergeometric testing was further applied on each subset of differentially expressed genes to determine significantly overrepresented key cellular processes (corrected p-value < 0.01) (S2 Dataset). P-values were adjusted by Benjamini-Hochberg correction for multiple testing (24).

**REFERENCES**

1. Eissing T, Kuepfer L, Becker C, Block M, Coboeken K, Gaub T, et al. A computational systems biology software platform for multiscale modeling and simulation: integrating whole-body physiology, disease biology, and molecular reaction networks. Front Physiol. 2011;2:4.

2. Willmann S, Solodenko J, Sevestre M, Lippert J, Schmitt W. A pharmacodynamic extension for the physiology-based pharmacokinetic whole-body model PK-Sim((R)). Eur J Pharm Sci. 2004;23:S75–S75.

3. Willmann S, Lippert J, Schmitt W. From physicochemistry to absorption and distribution: predictive mechanistic modelling and computational tools. Expert Opin Drug Metab Toxicol. 2005 Jun;1(1):159–68.

4. Willmann S, Lippert J, Sevestre M, Solodenko J, Fois F, Schmitt W. PK-Sim®: a physiologically based pharmacokinetic “whole-body” model. Biosilico. 2003 Sep;1(4):121–4.

5. Kuepfer L, Niederalt C, Wendl T, Schlender J, Willmann S, Lippert J, et al. Applied Concepts in PBPK modeling: How to build a PBPK/PD model. CPT Pharmacometrics Syst Pharmacol. 2016 Sep;

6. Metsalu T, Vilo J. ClustVis: a web tool for visualizing clustering of multivariate data using Principal Component Analysis and heatmap. Nucleic Acids Res. 2015;43(W1):W566-570.

7. Hoofnagle JH, Serrano J, Knoben JE, Navarro VJ. LiverTox: a website on drug-induced liver injury. Hepatology. 2013 Mar;57(3):873–4.

8. Clemedson C, Kolman A, Forsby A. The integrated acute systemic toxicity project (ACuteTox) for the optimisation and validation of alternative in vitro tests. Altern Lab Anim. 2007 Mar;35(1):33–8.

9. Gregoriano C, Ceschi A, Rauber-Lüthy C, Kupferschmidt H, Banner NR, Krähenbühl S, et al. Acute thiopurine overdose: Analysis of reports to a national poison centre 1995-2013. PLoS One. 2014 Jan;9(1):e86390.

10. Tenenbein MSM, Tenenbein MSM. Acute pancreatitis due to erythromycin overdose. Pediatr Emerg Care. 2005 Oct;21(10):675–6.

11. Aguiar Bujanda D, Cabrera Suárez MÁA, Bohn Sarmiento U, Aguiar Morales J. Successful recovery after accidental overdose of cyclophosphamide. Ann Oncol. 2006 Aug;17(8):1334.

12. Spalding CT, Buss WC. Toxic overdose of isoniazid, rifampicin and ethambutol. Eur J Clin Pharmacol. 1986 Jan;30(3):381–2.

13. von Mach M a, Hermanns-Clausen M, Koch I, Hengstler JG, Lauterbach M, Kaes J, et al. Experiences of a poison center network with renal insufficiency in acetaminophen overdose: an analysis of 17 cases. Clin Toxicol (Phila). 2005;43(1):31–7.

14. Igarashi Y, Nakatsu N, Yamashita T, Ono A, Ohno Y, Urushidani T, et al. Open TG-GATEs: a large-scale toxicogenomics database. Nucleic Acids Res. 2015 Jan 13;43(Database issue):D921-7.

15. Chen M, Vijay V, Shi Q, Liu Z, Fang H, Tong W. FDA-approved drug labeling for the study of drug-induced liver injury. Drug Discov Today. Elsevier Ltd; 2011 Aug;16(15–16):697–703.

16. Herpers B, Wink S, Fredriksson L, Di Z, Hendriks G, Vrieling H, et al. Activation of the Nrf2 response by intrinsic hepatotoxic drugs correlates with suppression of NF-κB activation and sensitizes toward TNFα-induced cytotoxicity. Arch Toxicol. 2015 May 31;90(5):1163–79.

17. Skrbo A, Begović B, Skrbo S. [Classification of drugs using the ATC system (Anatomic, Therapeutic, Chemical Classification) and the latest changes]. Med Arh. 2004;58(1 Suppl 2):138–41.

18. Benet LZ. The role of BCS (biopharmaceutics classification system) and BDDCS (biopharmaceutics drug disposition classification system) in drug development. J Pharm Sci. 2013 Jan;102(1):34–42.

19. Kasim N a, Whitehouse M, Ramachandran C, Bermejo M, Lennernäs H, Hussain AS, et al. Molecular properties of WHO essential drugs and provisional biopharmaceutical classification. Mol Pharm. 2004;1(1):85–96.

20. Value SJRI, Samy WM. Class II drugs; a dissolution / bioavailability challenge: Flutamide-loaded spray dried lactose for dissolution control. Int J Drug Dev Res. 2012;4(2):195–204.

21. Wu Z, Irizarry RA, Gentleman R, Martinez-Murillo F, Spencer F. A Model-Based Background Adjustment for Oligonucleotide Expression Arrays. J Am Stat Assoc. 2004 Dec;99(468):909–17.

22. Dai M, Wang P, Boyd AD, Kostov G, Athey B, Jones EG, et al. Evolving gene/transcript definitions significantly alter the interpretation of GeneChip data. Nucleic Acids Res. 2005 Jan 27;33(20):e175.

23. Smyth GK. Linear models and empirical bayes methods for assessing differential expression in microarray experiments. Stat Appl Genet Mol Biol. 2004 Jan 12;3(1):Article3.

24. Benjamini Y, Hochberg Y. Controlling the false discovery rate: A practical and powerful approach to multiple testing. J R Stat Soc Ser B. 1995;57(1):289–300.
